# Resolution of Pulmonary Inflammation Induced by Carbon Nanotubes and Fullerenes in Mice: Role of Macrophage Polarization

**DOI:** 10.3389/fimmu.2020.01186

**Published:** 2020-06-12

**Authors:** Chol Seung Lim, Dale W. Porter, Marlene S. Orandle, Brett J. Green, Mark A. Barnes, Tara L. Croston, Michael G. Wolfarth, Lori A. Battelli, Michael E. Andrew, Donald H. Beezhold, Paul D. Siegel, Qiang Ma

**Affiliations:** ^1^Receptor Biology Laboratory, Toxicology and Molecular Biology Branch, Health Effects Laboratory Division, National Institute for Occupational Safety and Health, Centers for Disease Control and Prevention, Morgantown, WV, United States; ^2^Pathology and Physiology Research Branch, Health Effects Laboratory Division, National Institute for Occupational Safety and Health, Centers for Disease Control and Prevention, Morgantown, WV, United States; ^3^Allergy and Clinical Immunology Branch, Health Effects Laboratory Division, National Institute for Occupational Safety and Health, Centers for Disease Control and Prevention, Morgantown, WV, United States; ^4^Bioanalytics Branch, Health Effects Laboratory Division, National Institute for Occupational Safety and Health, Centers for Disease Control and Prevention, Morgantown, WV, United States; ^5^Office of the Director, Health Effects Laboratory Division, National Institute for Occupational Safety and Health, Centers for Disease Control and Prevention, Morgantown, WV, United States

**Keywords:** pulmonary inflammation, resolution, MWCNT, C60F, SPM, macrophage polarization, ALOX5AP, ALOX15

## Abstract

Pulmonary exposure to certain engineered nanomaterials (ENMs) causes chronic lesions like fibrosis and cancer in animal models as a result of unresolved inflammation. Resolution of inflammation involves the time-dependent biosynthesis of lipid mediators (LMs)—in particular, specialized pro-resolving mediators (SPMs). To understand how ENM-induced pulmonary inflammation is resolved, we analyzed the inflammatory and pro-resolving responses to fibrogenic multi-walled carbon nanotubes (MWCNTs, Mitsui-7) and low-toxicity fullerenes (fullerene C60, C60F). Pharyngeal aspiration of MWCNTs at 40 μg/mouse or C60F at a dose above 640 μg/mouse elicited pulmonary effects in B6C3F1 mice. Both ENMs stimulated acute inflammation, predominated by neutrophils, in the lung at day 1, which transitioned to histiocytic inflammation by day 7. By day 28, the lesion in MWCNT-exposed mice progressed to fibrotic granulomas, whereas it remained as alveolar histiocytosis in C60F-exposed mice. Flow cytometric profiling of whole lung lavage (WLL) cells revealed that neutrophil recruitment was the greatest at day 1 and declined to 36.6% of that level in MWCNT- and 16.8% in C60F-treated mice by day 7, and to basal levels by day 28, suggesting a rapid initiation phase and an extended resolution phase. Both ENMs induced high levels of proinflammatory leukotriene B4 (LTB4) and prostaglandin E2 (PGE2) with peaks at day 1, and high levels of SPMs resolvin D1 (RvD1) and E1 (RvE1) with peaks at day 7. MWCNTs and C60F induced time-dependent polarization of M1 macrophages with a peak at day 1 and subsequently of M2 macrophages with a peak at day 7 in the lung, accompanied by elevated levels of type 1 or type 2 cytokines, respectively. M1 macrophages exhibited preferential induction of arachidonate 5-lipoxygenase activating protein (ALOX5AP), whereas M2 macrophages had a high level expression of arachidonate 15-lipoxygenase (ALOX15). Polarization of macrophages *in vitro* differentially induced ALOX5AP in M1 macrophages or ALOX15 in M2 macrophages resulting in increased preferential biosynthesis of proinflammatory LMs or SPMs. MWCNTs increased the M1- or M2-specific production of LMs accordingly. These findings support a mechanism by which persistent ENM-induced neutrophilic inflammation is actively resolved through time-dependent polarization of macrophages and enhanced biosynthesis of specialized LMs via distinct ALOX pathways.

## Introduction

Engineered nanomaterials (ENMs) have been used for a wide range of industrial, commercial, and biomedical applications (1, 2). ENMs, such as carbon nanotubes (CNTs), are nano-scaled in at least one dimension and exhibit unique physicochemical properties. While many of these properties are targets of interest for technological innovations and industrial use, some properties are associated with the adverse effects of toxic particles and fibers, such as persistence in the air once aerosolized, large surface area, poor solubility, and resistance to degradation in biological systems (3, 4). Certain ENMs, such as filamentous multi-walled CNTs (MWCNTs), can cause acute inflammation and chronic pulmonary lesions, including granulomatous inflammation, interstitial fibrosis, lung cancer, and mesothelioma (4–9). The early phase pulmonary response to CNTs is characterized by acute inflammation and rapid onset of fibrotic changes that may progress to chronic fibrosis in the lung (10–12). In these observations, acute inflammation precedes chronic fibrotic changes, suggesting transitional events between inflammation and tissue fibrosis, namely, resolution of inflammation and programmatic repair, that may underlie the development of lung fibrosis (4, 12, 13). How ENM-induced pulmonary inflammation is resolved and whether the resolution or a “failed” resolution contributes to the pathogenesis of chronic phenotypes induced by ENMs and other particulates have not been investigated.

Acute inflammation is a host defense mechanism against infectious or sterile tissue damage with a physiological purpose to restore tissue homeostasis (14, 15). The molecular and cellular events in inflammation are characterized by increased blood flow, leukocyte infiltration, and production of protein and chemical mediators (16). However, uncontrolled or unresolved inflammation can lead to excessive tissue damage and loss of organ functions (17, 18). Persistent, unresolved inflammation is not only a typical sign of classical inflammatory diseases, such as asthma, rheumatoid arthritis, and tuberculosis, but also an underlying feature of a variety of human disorders not previously thought to have an inflammatory component, including atherosclerosis, cardiovascular disease, and cancer (19–23). Recent studies suggest that ineffective resolution of inflammation contributes to the pathogenesis of lung diseases, including chronic obstructive pulmonary disease and pulmonary fibrosis (24, 25).

An effective resolution program may help prevent the progression from acute inflammation to chronic lesions. Initiation of acute inflammation and its resolution is highly regulated by potent lipid mediators (LMs). These LMs specifically promote the initiation, self-resolving or, when unresolved, chronic progression, of inflammation in a time- and context-dependent manner (18, 26). Prostaglandins (PGs) and leukotrienes (LTs) are biosynthesized from arachidonic acid (AA) via pathways of cyclooxygenase (COX) and arachidonate 5-lipoxygenase (ALOX5) in mice, respectively (27). These LMs elicit acute inflammation by inducing dilation of blood vessels and extravasation and activation of leukocytes, which include mainly neutrophils, but also monocytes, other polymorphonuclear leukocytes (PMNs), and lymphocytes (26). Upon activation of acute inflammation, a different type of small LMs, termed specialized pro-resolving mediators (SPMs), are temporally produced to actively promote the resolution of inflammation and prevent the progression of inflammation (18, 28). SPMs consist of several groups of LMs, including the D-series resolvins (RvDs), protectins, and maresins derived from docosahexaenoic acid (DHA), the E-series resolvins (RvEs) from eicosapentaenoic acid (EPA), and lipoxins (LXs) from AA. These molecules are biosynthesized via the activation of arachidonate 15-lipoxygenase (ALOX15)-dependent pathways (29). SPMs have dual anti-inflammatory and pro-resolving properties (30, 31). Dysregulation of SPM production or failure of controlled resolution may result in chronic inflammation, which constitutes a major component of many human diseases including lung fibrosis and cancer.

One key cellular event in the resolution of acute inflammation is the depletion of neutrophils from local inflamed tissue, mediated through reduction of leukocyte infiltration and induction of programmed death, such as apoptosis, of neutrophils, followed by removal of dead or injured cells (32). Clearance of apoptotic neutrophils by phagocytosis is known as efferocytosis. Macrophages are actively involved in the clearance of pathogenic microorganisms and non-biological particles, as well as apoptotic leukocytes and damaged tissue cells and, therefore, are a central component in the resolution of acute inflammation (33–35). Recent studies have highlighted the importance of specific subsets or reprogramming states of macrophages during inflammation initiation and progression (36, 37). In this context, the M1 (classically activated or polarized) macrophages generate proinflammatory LMs, including leukotriene B4 (LTB4) and prostaglandin E2 (PGE2), whereas M2 (alternatively activated or polarized) macrophages produce predominantly pro-resolving LMs, i.e., SPMs like resolvins and LXs, to promote efferocytosis, tissue regeneration, and return of homeostasis (38, 39). We have previously shown that MWCNTs stimulate the M1 and M2 polarization of macrophages in exposed lungs to regulate the initiation and resolution of acute inflammation, which, nonetheless, leads to chronic progression to fibrosis. This dynamic evolvement from proinflammatory to pro-resolving but ultimately profibrotic development likely reflects a time-dependent progression from type 1 to type 2 inflammation prior to progression to fibrosis (12, 13, 35, 40–42). Whether pulmonary exposure to CNTs and other ENMs alters the pathways for the synthesis of proinflammatory LMs and SPMs and, if so, whether CNT-induced M1 and M2 polarization affects the production of SPMs and resolution of inflammation in the lung, have not been examined. ENMs vary considerably in their physicochemical properties and pathologic effects. In this connection, it is of interest to examine if chemically similar ENMs with dissimilar dimensions and fibrogenicity differ in their inflammatory and resolution activities.

In this study, we examined how ENM-induced pulmonary inflammation is resolved. To achieve this goal, we analyzed and compared the inflammatory and pro-resolving responses to fiber-like, potently toxic MWCNTs (i.e., Mitsui-7) and spherical, low-toxicity fullerenes (i.e., C60F) in mouse lungs. Both ENMs are pure carbon-based and, therefore, are chemically alike to each other. Yet, the ENMs differ substantially in shape and exhibit vastly different potencies in their toxicity. MWCNTs at a low dose (40 μg/mouse) and C60F at a high dose (above 640 μg/mouse) stimulated rapid neutrophilic inflammation in the lung, which was reduced in intensity 1 week after exposure but, nonetheless, progressed to fibrotic granulomas in MWCNT-exposed lungs by day 28 post-exposure. Both ENMs induced the production of proinflammatory LMs that peaked at day 1 and SPMs that reached their highest levels at day 7, as measured in whole lung lavage (WLL) fluids. MWCNTs and C60F elicited time-dependent polarization and accumulation of M1 macrophages with a peak at day 1 and M2 macrophages with a peak at day 7, accompanied by elevated levels of type 1 or type 2 cytokines, respectively. Moreover, M1 macrophages exhibited preferential induction of ALOX5 activating protein (ALOX5AP), whereas the M2 macrophages had high level expression of ALOX15. Polarization of M1 and M2 macrophages *in vitro* differentially induced the expression of ALOX5AP in M1 macrophages or ALOX15 in M2 macrophages resulting in differential biosynthesis of proinflammatory LMs or SPMs from endogenous substrates, which was enhanced by MWCNTs. These results suggest a mechanism for the resolution of pulmonary inflammation in response to ENMs. In this model, low dose MWCNT or high dose C60F exposure induces time-dependent polarization of macrophages and enhances the biosynthesis of specialized LMs via activation of ALOX pathways associated with M1-M2 macrophage phenotypes. In turn, these cellular and molecular events orchestrate a prolonged resolution of pulmonary inflammation in the continued presence of ENMs. While the vast difference in potency between MWCNTs and C60F remains an enigma, these findings provide a new framework for mechanistic analysis of resolution of lung inflammation induced by ENMs and other inhaled particulates relating to fibrosis development.

## Materials and Methods

### Characterization and Preparation of MWCNTs and C60F

The MWCNTs used in this study were obtained from Mitsui & Company (Mitsui-7, XNRI 1, lot #-0507 2001K28, Tokyo, Japan). C60F was purchased from Sigma Aldrich (St. Louis, MO, USA). Characterization of MWCNTs and C60F was performed using transmission electron microscopy (TEM). A sample of MWCNTs and C60F were suspended in isopropanol, sonicated, and dispersed onto a TEM grid with a carbon film. For MWCNTs, length measurements were taken from the longest straight distance between two points. The width measurement was the distance perpendicular to the structural walls of the CNTs. To determine C60F diameter, two perpendicular measurements were collected on each particle. Morphology of C60F was further examined using field emission scanning electron microscopy (FESEM).

A dispersion medium (DM; 0.9% saline supplemented with 5.5 mM D-glucose, 0.6 mg/ml mouse serum albumin, and 0.01 mg/ml 1,2-dipalmitoyl-sn-glycero-3-phosphocholine) was modified from one previously developed and validated by our laboratory as a vehicle for nanotoxicology studies (43), and was used to prepare suspensions of MWCNTs and C60F following a two-step dispersion procedure (11).

### Animals and Treatment

Six-week old male B6C3F1 mice were purchased from the Jackson Laboratory (Bar Harbor, ME, USA). Mice were maintained in an accredited, specific pathogen-free and environmentally controlled facility at the National Institute for Occupational Safety and Health. All animals received humane care and all experiments involving animals were approved by the Institutional Animal Care and Use Committee. Ten mice per treatment at each timepoint were treated with a single dose of 50 μl of DM, MWCNTs (40 μg/mouse), or C60F (640 or 1,280 μg/mouse) in suspensions by pharyngeal aspiration as described elsewhere (4, 11). A saline control was used to establish baseline levels of the measurements. At day 1, 7, or 28 post-exposure, the mice were euthanized with an intraperitoneal injection of a sodium pentobarbital euthanasia solution (100–300 mg/kg body weight; Zoetis, Florham Park, NJ, USA), followed by exsanguination once the mouse was unresponsive to a toe pinch, for molecular, immunologic, and pathological examinations.

Given the apparent paucity of data concerning human exposure to ENMs, the MWCNT and C60F doses chosen for the mouse study were estimated to approximate human occupational exposure. A recent study reported peak MWCNT-containing airborne dust levels of approximately 10.6 μg MWCNTs/m^3^ among workers exposed to MWCNTs in eight U.S. manufacturing facilities (44). Airborne particle concentrations of carbonaceous nanomaterials, including fullerenes, was measured in a commercial nanotechnology facility (45). Fine particle mass concentrations were measured in three different areas, ranged from 50 to 125 μg per m^3^. Assuming a worker performs light work in an environment with MWCNT aerosol of 10.6 μg/m^3^ or C60F aerosol of 50 μg/m^3^, and has a minute ventilation of 20 L/min (46) with a deposition fraction of 30% (47) and an alveolar epithelium surface area of 102 m^2^ (48), the estimated human exposure per year would be 7.3 mg/m^2^ alveolar epithelium for MWCNTs and 34.6 mg/m^2^ alveolar epithelium for C60F. Therefore, a 40 μg MWCNT exposure in mouse approximates human deposition for a person performing light work for 11 years and a 1,280 μg C60F exposure in mouse for 9 months.

### Macrophage Culture, Polarization, and Treatment

The J774A.1 murine monocyte/macrophage cell line was purchased from American Type Culture Collection (TIB-67, ATCC, Manassas, VA, USA). The cells were grown in the Dulbecco's Modified Eagle's Medium (DMEM) with 10% fetal bovine serum (FBS) (both from Thermo Fisher Scientific, Waltham, MA, USA). For differential polarization of M1 and M2 macrophages, cells at a density of 5 × 10^5^ cells/ml were seeded in DMEM with 3% FBS for 1 day. M1 polarization was then induced by incubation with interferon γ (IFN-γ, Sigma Aldrich) at 20 ng/ml plus lipopolysaccharides (LPS, Sigma Aldrich) at 100 ng/ml for indicated time (typically 3 days). M2 polarization was induced by incubation of the cells with interleukin (IL) 4 (Sigma Aldrich) at 20 ng/ml for indicated time (typically 3 days). Polarized M1 or M2-macrophages were treated with DM or MWCNTs at 2.5 μg/ml for indicated time (i.e., 1, 2, or 3 days post-polarization), before the cells were examined for protein expression and production of LMs.

### Whole Lung Lavage (WLL) Preparation

After 1, 7, or 28 days post-exposure, mice were euthanized by intraperitoneal injection of a sodium pentobarbital euthanasia solution, followed by exsanguination, as described under Animals and Treatment. A tracheal cannula was inserted and WLL was performed through the cannula using ice cold Ca^2+^- and Mg^2+^-free phosphate buffered saline (PBS), pH 7.4. The first lavage (0.6 ml) was kept separated from the rest of the lavage fluid. Subsequent lavages, each with 1 ml of PBS, were performed until a total of 3 ml of lavage fluid was collected. WLL cells were isolated by centrifugation (650×g, 5 min, 4°C). An aliquot of the acellular supernatant from the first WLL was decanted, transferred to tubes, and stored at −80°C for later analyses. The acellular supernatants from the remaining lavage samples were decanted and discarded. WLL cells isolated from the first and subsequent lavages from the same mouse were pooled after resuspension in PBS, centrifuged a second time (650×g, 5 min, 4°C), and the supernatant decanted and discarded. The WLL cell pellet was then resuspended in PBS and placed on ice. Total WLL cell counts were obtained using a Coulter Multisizer 3 (Coulter Electronics, Hialeah, FL, USA) and cell differentials were determined by flow cytometry.

### Enhanced Darkfield Microscopy

ENMs, such as CNTs, have dimensions less than the wavelength of light and closely packed atoms and, hence, a refractive index that is significantly different from those of the environment, such as biologic tissues and the mounting medium. These characteristics of ENMs produce significantly greater scattering of light than by the surrounding tissues. The enhanced-darkfield optical system images light scattered in tissue sections and ENMs in tissues would stand out from the surrounding tissues with a high contrast.

To visualize ENMs and their deposition in lung tissue, formalin-fixed lung sections were examined under an enhanced darkfield microscope as described previously (49). Using this method of imaging, it is practical to scan whole lung sections at a relatively low magnification (40–60× objectives) to identify ENMs that would not be detected by other means. The optical system consists of high signal-to-noise, darkfield-based illumination optics adapted to an Olympus BX-41 microscope (CytoViva, Auburn, AL, USA). Sections for dark-field examination were specifically cut from paraffin blocks and collected on ultrasonically cleaned, laser cut slides (Schott North America Inc, Elmsford, NY, USA) to avoid nanoparticle contamination from the ground edges of traditional glass slides. After staining with Picrosirius red-hematoxylin, the sections were cover-slipped with Permount (Vector Laboratories, Inc., Burlingame, CA, USA). After alignment of the substage oil immersion optics with a 10× objective, sections were examined with 60× air or 100× oil immersion objectives. Enhanced darkfield images were taken at 2400×4800 pixels with an Olympus DP-73 digital camera (Olympus America Inc., Center Valley, PA, USA).

### Histopathology

For histopathological examination, 3 mice per treatment group, separate from those used for WLL studies, were euthanized by intraperitoneal injection of sodium pentobarbital euthanasia solution, followed by exsanguination, as described under Animals and Treatment. The lung was removed, fixed with 10% neutral buffered formalin by intratracheal perfusion, and embedded in paraffin. Sections of 5 μm thickness were subjected to hematoxylin and eosin (H&E) staining or Picrosirius red staining following standard procedures.

### Immune Cell Profiling by Flow Cytometry

Cells were recovered from WLL fluids by centrifugation (400×g, 5 min at 4°C). Cells were resuspended in 1× PBS with normal rat sera (Sigma Aldrich) and stained using rat anti-mouse CD16/32 (clone 2.4G2, BD Biosciences, San Jose, CA, USA) for 5 min at room temperature. Cells were then stained with the following fluorescent antibody cocktail in a fluorescence-activated cell sorting (FACS) buffer (1× PBS, 5% BSA, 2 mM EDTA) for 25 min at 4°C: anti-CD103 (clone M290), anti-CD19 (clone 1D3), anti-Siglec F (clone E50-2440) (BD Biosciences), and anti-CD11b (clone M1/70), anti-CD11c (clone N418), anti-CD3 (clone 17A2), anti-CD49b (clone DX5), anti-Ly6C (HK1.4), anti-Ly6G (1A8) (Biolegend, San Diego, CA, USA). After washing with 2 ml of the FACS buffer, cells were centrifuged (400×g, 5 min at 4°C). Cells were fixed in the BD Cytofix Fixation Buffer (BD Biosciences) for 10 min at room temperature, then washed with 2 ml of FACS buffer and centrifuged. Cells were resuspended in 250 μl of FACS buffer and more than 10,000 events were acquired using LSR II flow cytometer (BD Biosciences). Data were analyzed using FlowJo software (FlowJo LLC, Ashland, Oregon, USA). Live cells were discriminated from dead cells, doublets, and debris first using forward scatter (FSC)-A vs. side scatter (SSC)-A (**Figure 5A**a), then gating upon singlet populations in FSC-H vs. FSC-W view (**Figure 5A**b), then SSC-H vs. SSC-W view (**Figure 5A**c). Innate immune cells (**Figure 5A**d) and lymphocytes (**Figure 5A**e) were subsequently identified based on the cell surface markers indicated on the X and Y axes of the flow cytometry pictographs. Cell populations were gated sequentially on populations negative for the previous populations as follows: alveolar macrophages, eosinophils, neutrophils, lymphocytes, dendritic cells (DCs), and monocytes.

### Cytokine Measurement

Measurement of cytokines in WLL fluids and lung tissue extract was performed by multiplex immunoassay using ProcartaPlex mouse cytokine/chemokine 36-plex kit (Thermo Fisher Scientific) on a Luminex 200 instrument system equipped with xPONENT software (Thermo Fisher Scientific) following the manufacturer's instructions. Twenty-five microliter of WLL fluids or lung tissue homogenates in 1x PBS containing 1x protease inhibitor cocktail (Thermo Fisher) was analyzed to determine the concentration of each cytokine. For lung tissue, concentrations of cytokines were calculated and expressed as pg/mg protein.

### Enzyme-Linked Immunosorbent Assay (ELISA)

Proinflammatory LMs (LTB4, PGE2) or SPMs (RvD1, LXA4, RvE1) were detected in WLL fluids collected from mouse lungs exposed to saline, DM, MWCNTs, or C60F for the indicated dose and days using ELISA. All ELISA kits were purchased from MyBioSource (San Diego, CA, USA) and measurement was performed by following the manufacturer's protocol using a microplate reader equipped with SOFTmax PRO 4.0 (Molecular Devices, Sunnyvale, CA). To detect and quantify SPMs released from polarized M1 or M2-macrophages *in vitro*, cell-free media from cultures of M1 or M2 macrophages were collected and used for quantification of LTB4, PGE2, and RvD1 using ELISA.

### Immunofluorescent Staining and Confocal Microscopy

Formalin-fixed, paraffin-embedded lung tissue sections (left lung lobe, 5 μm) were deparaffinized, antigen-unmasked by heating tissue sections in universal antigen retrieval reagent (Cell Signaling Technology, Danvers, MA), and used to perform immunofluorescent staining. The primary antibodies used were rabbit anti-F4/80 (1:100, Thermo Fisher Scientific) or mouse anti-F4/80 (1:100, Santa Cruz Biotechnology, Santa Cruz, CA), mouse anti-CD68 (1:100, Novus Biologicals, Centennials, CA), mouse anti-CD206 (1:100, Abcam, Cambridge, MA, USA), rabbit anti-ALOX5AP (1:100, Abcam), and mouse anti-ALOX15 (1:200, Abcam) antibodies. Images were taken in three to five randomly selected fields per lung slice, three lung slices per mouse, using a Zeiss LSM 780 confocal microscope with a 63× magnification lens (Carl Zeiss Microscopy, Jena, Germany). More than 500 cells from captured microscopic images per each treatment were counted using the ImageJ software (NIH, Bethesda, USA) and the number of cells with double positive staining per every 100 cells were presented as mean ± SEM (*n* = 3).

### Immunoblotting

J774A.1 cells (a mouse macrophage cell line) were treated as indicated and the cells were lysed in a lysis buffer (10 mM Tris, pH 7.4, 1% SDS) with 1× proteinase inhibitor cocktail (Thermo Fisher). Cell lysates were collected and sonicated for 10 s. The supernatant was collected and lysate proteins (20 μg each sample) were analyzed on 8, 10, or 12% SDS-PAGE gel and transferred onto nitrocellulose membrane. The membrane was incubated with 5% non-fat dry milk in Tris-buffered saline with 0.05% Tween 20 for 1 h at room temperature to block non-specific binding, before incubation with primary antibodies. The primary antibodies used were mouse anti-CD68 (1:200, Novus Biologicals), mouse anti-CD86 (1:200, Novus Biologicals), rabbit anti-CD163 (1:250, Abcam), mouse anti-CD206 (1:250, Abcam), rabbit anti-ALOX5 (1:1,000, Abcam), mouse anti-ALOX15 (1:500, Abcam), rabbit anti-ALOX5AP (1:500, Abcam), or mouse anti-β-actin (1:4,000, Sigma Aldrich) antibodies. After incubation with horseradish peroxidase-conjugated goat anti-mouse or goat anti-rabbit IgG (both from Jackson ImmunoResearch laboratories, West Grove, PA; 1:5,000), immunoreactive bands were developed with Enhanced chemiluminescence (Thermo Fisher Scientific) on X-ray films. Film images were scanned using HP scanjet (Hewlett-Packard, Palo Alto, CA, USA) and were used to quantify each band with ImageJ software (NIH) with normalization to β-actin level.

### Statistical Analysis

For statistical analysis of multiple samples, such as days post-exposure, one-way analysis of variance (ANOVA) was performed, followed by Tukey's test for comparison between two samples, using the GraphPad Prism 8 software (GraphPad, San Diego, CA, USA). Statistical analyses of differences between two groups were also performed using two-tailed Student's *t-*test. Data were presented as means ± SEM. A *p-*value of <0.05 was considered statistically significant as indicated for individual experiment. ^*^p < 0.05; ^**^p < 0.01; and ^***^p < 0.001.

## Results

### Characterization of ENMs

To study the resolution of ENM-induced pulmonary inflammation, we characterized and compared the inflammatory and pro-resolving responses during the development of fibrosis following exposure to MWCNTs (Mitsui-7) and C60F. The MWCNTs have a fiber-like shape with high rigidity and are highly toxic and fibrogenic in rodent lungs upon pulmonary exposure, thus representing a highly toxic ENMs (4, 11). C60F are spherical ENMs and cause certain pulmonary effects upon inhalation exposure (50). Therefore, these carbon-based ENMs are chemically similar but differ considerably in their dimensions and possibly their toxic effects in the lung.

**Figure 1:**
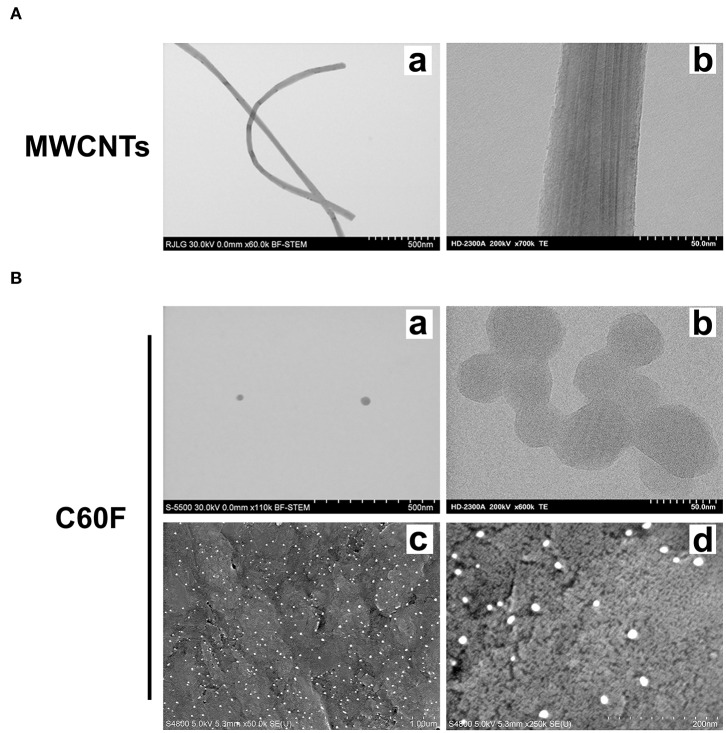
Morphological characterization of MWCNTs and C60F. **(A)** Morphology of MWCNTs. A representative low (a) or high (b) magnification TEM micrograph of MWCNTs showing the distinctive fiber-like shape and multi-walled structure of MWCNTs. **(B)** Morphology of C60F. For (a) and (b), a representative low (a) or high (b) magnification TEM micrograph of the C60F was shown demonstrating their distinctive spherical morphology. For (c) and (d), FESEM micrographs of C60F at a low (c) or high (d) magnification were shown confirming the spherical morphology and even distribution.

We first characterized the morphology of the ENMs. Examination at lower magnifications of TEM for MWCNTs demonstrated their fiber-like shape (Figure 1Aa), while high resolution showed their distinctive multi-walled structure (Figure 1Ab). MWCNT length distribution was log normal with a mean of 4.46 μm and the 95% confidence interval (CI) of 4.08–4.88 μm; width distribution was normally distributed with a mean of 58.5 nm and the 95% CI of 56.0–61.0 nm. C60F at lower magnifications had the expected spherical morphology and had ordered carbon structure under examination at a high magnification (Figure 1Ba,b). The C60F diameter was log normal with a mean of 41.6 nm and the 95% CI of 40.1–43.2 nm. FESEM examination of C60F confirmed their spherical morphology and even distribution in suspension preparation (Figure 1Bc,d).

### Acute and Chronic Lesions Induced by Low Dose MWCNTs and High Dose C60F in Mouse Lungs

Adult B6C3F1 mice were exposed to MWCNTs or C60F by pharyngeal aspiration. An equal volume of saline was used to establish a baseline, whereas an equal volume of DM was used as a vehicle control. Mice were sacrificed for histopathological examination of lung sections at days 1, 7, and 28 post-exposure. These time points were chosen because the pulmonary response to MWCNTs at 40 μg at these time points reflects the initiation of acute inflammation, acute-to-chronic transition, and chronic and fibrotic progression, respectively (4, 11, 51). A preliminary dose-response study for C60F was carried out in a dose range from 40 to 2,560 μg, which identified a dose above 640 μg, including 1,280 μg, as being necessary for C60F to elicit evident inflammation in mouse lungs (data not shown). Comparison between the saline and DM groups did not show apparent differences in the microscopic appearance in the lung. Therefore, DM was used as control for further pathological examination.

**Figure 2:**
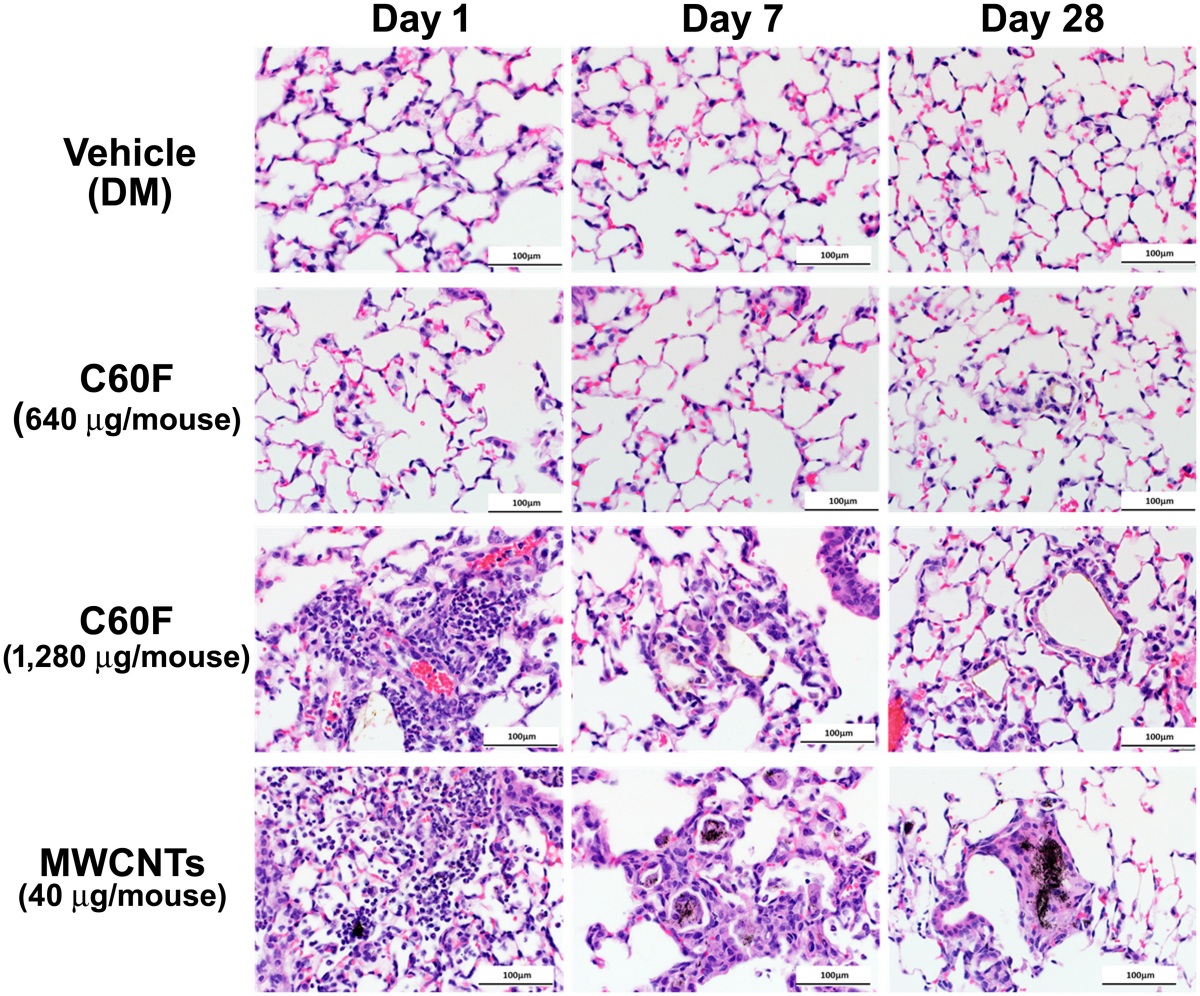
Histopathology of ENM-exposed mouse lungs. Adult male B6C3F1 mice were given DM, MWCNTs (40 μg), or C60F (640 or 1,280 μg) by pharyngeal aspiration and were euthanized at days 1, 7, and 28 post-exposure for histological examination of the lung. Representative photomicrographs of H&E stained sections of mouse lung demonstrating pathology following particle exposure (*n* = 3). Scale bar = 100 μm.

A separate set of mice were exposed to MWCNTs at 40 μg, or to C60F at 640 or 1,280 μg, and were sacrificed for histopathological examination of lung lesions at days 1, 7, and 28 post-exposure. Representative photomicrographs of lung pathology are shown in Figure 2. Examination of H&E stained lung slides revealed that at day 1 post-exposure, both ENMs (MWCNTs at 40 μg and C60F at 1,280 μg) induced acute inflammation that was multifocal in distribution and minimal or mild in severity for both particles. Inflammation was characterized by accumulations of neutrophils and macrophages at terminal bronchioles with extension into alveoli, often in association with deposits of foreign material. Similar acute inflammatory changes were not seen in lungs of mice exposed to either DM or to C60F at 640 μg dose (Figure 2). At day 7 post-exposure, resolution of acute neutrophilic inflammation with a concurrent shift to granulomatous inflammation was observed with both ENMs, i.e., MWCNTs at 40 μg and C60F at 1,280 μg. Microscopic changes in the lung at this time point included increased numbers of alveolar macrophages and accumulation of macrophages within alveolar walls (alveolar histiocytosis and interstitial inflammation) which was multifocally distributed and minimal or mild in severity. Progression to granulomas was seen at 28 days post-exposure to MWCNTs. This change was multifocal and mild in severity. In contrast, the primary microscopic change with exposure to C60F at both the 1,280 and 640 μg doses was multifocal, minimal alveolar histiocytosis. Similar chronic inflammatory changes were not seen in lungs of mice exposed to DM.

**Figure 3:**
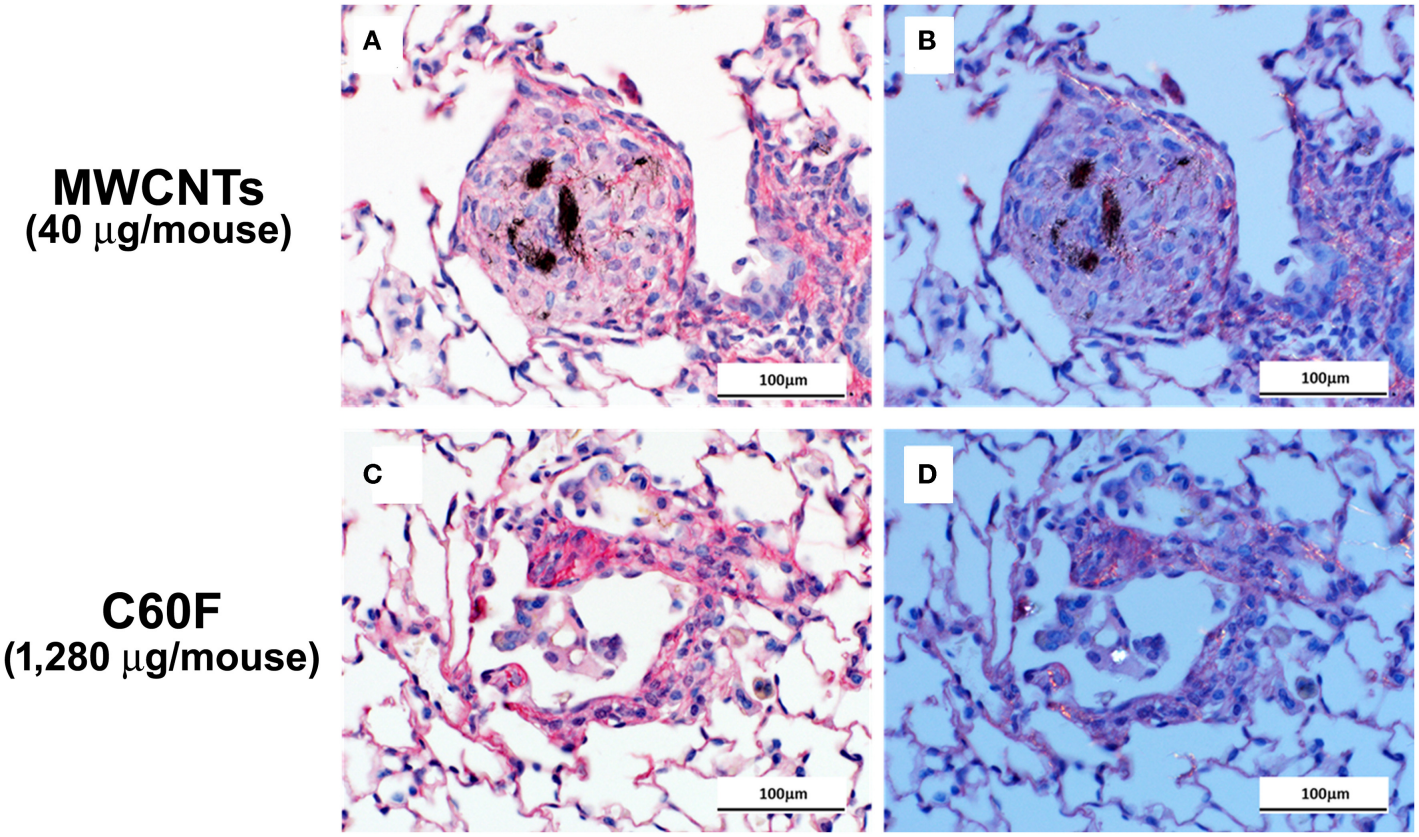
Bright field and polarizing light microscopy images of picrosirius red-stained sections of lung. Mice were treated as described in Figure 2 legend and the lungs were examined at 28 day post-exposure. **(A,C)** show red staining of extracellular fibrils within a granuloma and in a focus of chronic inflammation. Polarization confirms the presence of collagen in **(B,D)**. Scale bar = 100 μm.

Picrosirius red-stained slides were examined microscopically to evaluate for particle-induced pulmonary fibrosis at 28 days post-exposure. Fibrotic granulomas characteristic of MWCNT exposure in mice (11, 51) were seen in lung of exposed mice. However, this lesion was not a feature of C60F exposure at either dose tested. In rare instances, foci of minimal interstitial inflammation and fibrosis were seen in lung of C60F 1,280 μg exposed mice, suggesting the potential of this material to induce lung fibrosis at high doses (Figure 3). Fibrosis was not seen in lung of either DM or C60F (640 μg)-exposed mice.

**Figure 4:**
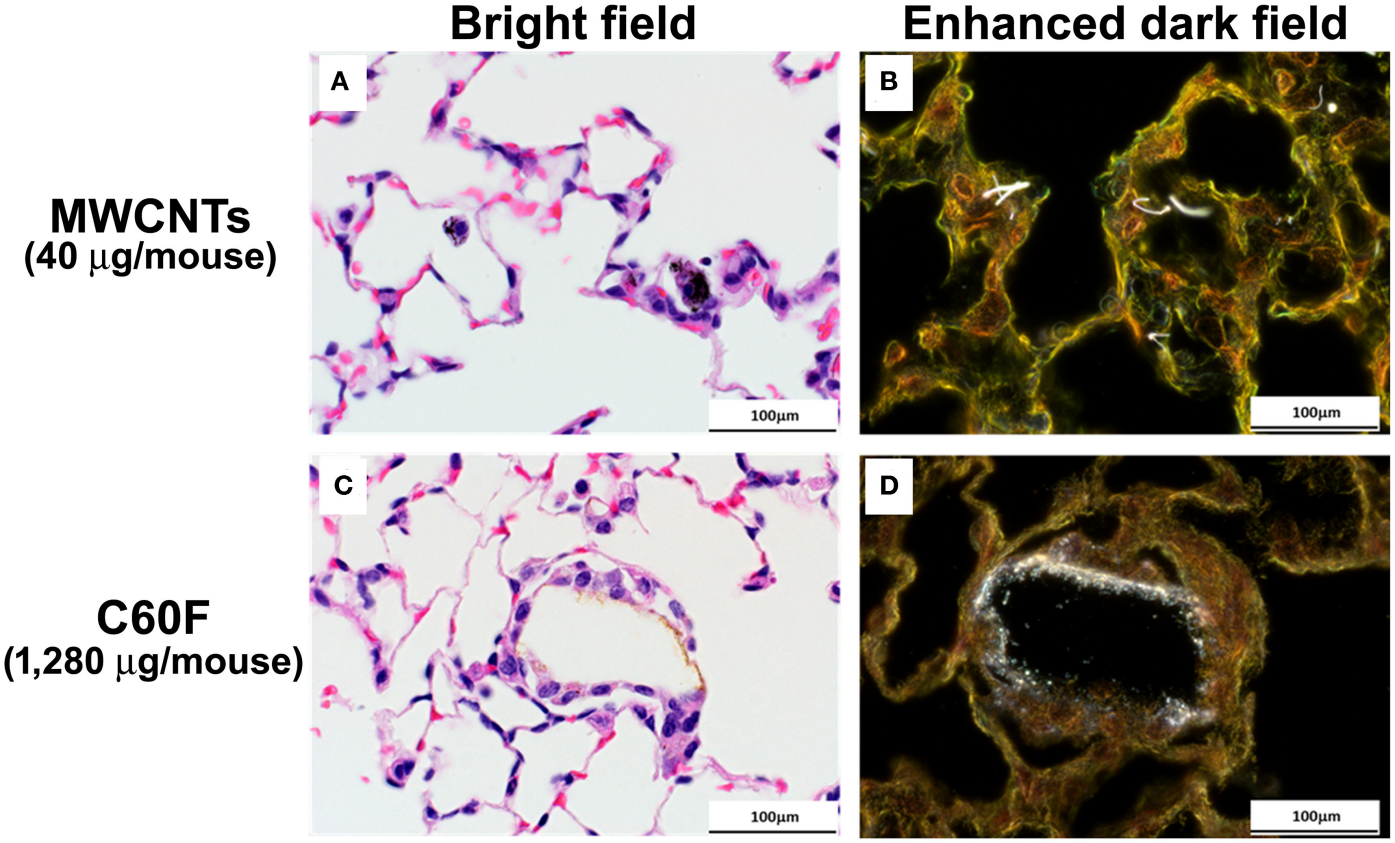
Bright field and enhanced dark field microscopy demonstrating the appearance of particles in sections of mouse lung. Mice were treated as described in Figure 2 legend and the lungs were examined at 28 days post-exposure. Representative photomicrographs of H&E stained sections are shown in **(A,C)**. MWCNT particles appears as black spicules. Agglomerated C60F appears as a thin rim of golden-brown material surrounded by macrophages. **(B,D)** are representative images of Picrosirius stained lung sections imaged using enhanced dark field microscopy, confirming the presence of nanoparticles within the sections (*n* = 3). Scale bar = 100 μm.

The appearance of MWCNTs in tissue sections at 28 day post-exposure has been well-described (49) with the material appearing with light microscopy as black spicule-shaped particles often within alveolar macrophages or in granulomas (Figure 4A). Although the diameter of C60F is below the resolution of light microscopy (~0.5 microns), microscopic examination of H&E stained lung sections often revealed golden brown, crystalline deposits of foreign material within alveoli often surrounded by a thin rim of macrophages, a finding consistent with agglomeration of the material *in vivo* (Figure 4C). Enhanced darkfield microcopy on Picrosirius red-stained slides confirmed the presence of nanoparticles within these deposits in exposed lung (Figures 4B,D).

Together, these results revealed that MWCNTs at a low dose and C60F at only a high dose stimulated rapid neutrophilic inflammation in the lung that was resolved to a large extent by 1 week after exposure but, nonetheless, evolved into fibrotic granulomas in MWCNT-exposed mice, and histiocytosis in high dose C60F-exposed mice. Inflammatory and fibrotic lesions were associated with retained particle deposits, suggesting a role for incomplete clearance in the progression from acute to chronic inflammation.

### Dynamic Infiltration of Immune Cells and Resolution of Neutrophilic Inflammation

Acute pulmonary inflammation is accompanied by prominent infiltration of leukocytes, mainly neutrophils, other PMNs, and monocytes, and secretion of inflammatory mediators into the alveolar space and airway lumen. These inflammatory events help eliminate ENMs at the airway and alveolar epithelial barrier surface and can be quantified in the WLL fluid. Therefore, we examined the dynamic change of immune cells in WLL fluids during acute and chronic responses using flow cytometric profiling.

**Figure 5:**
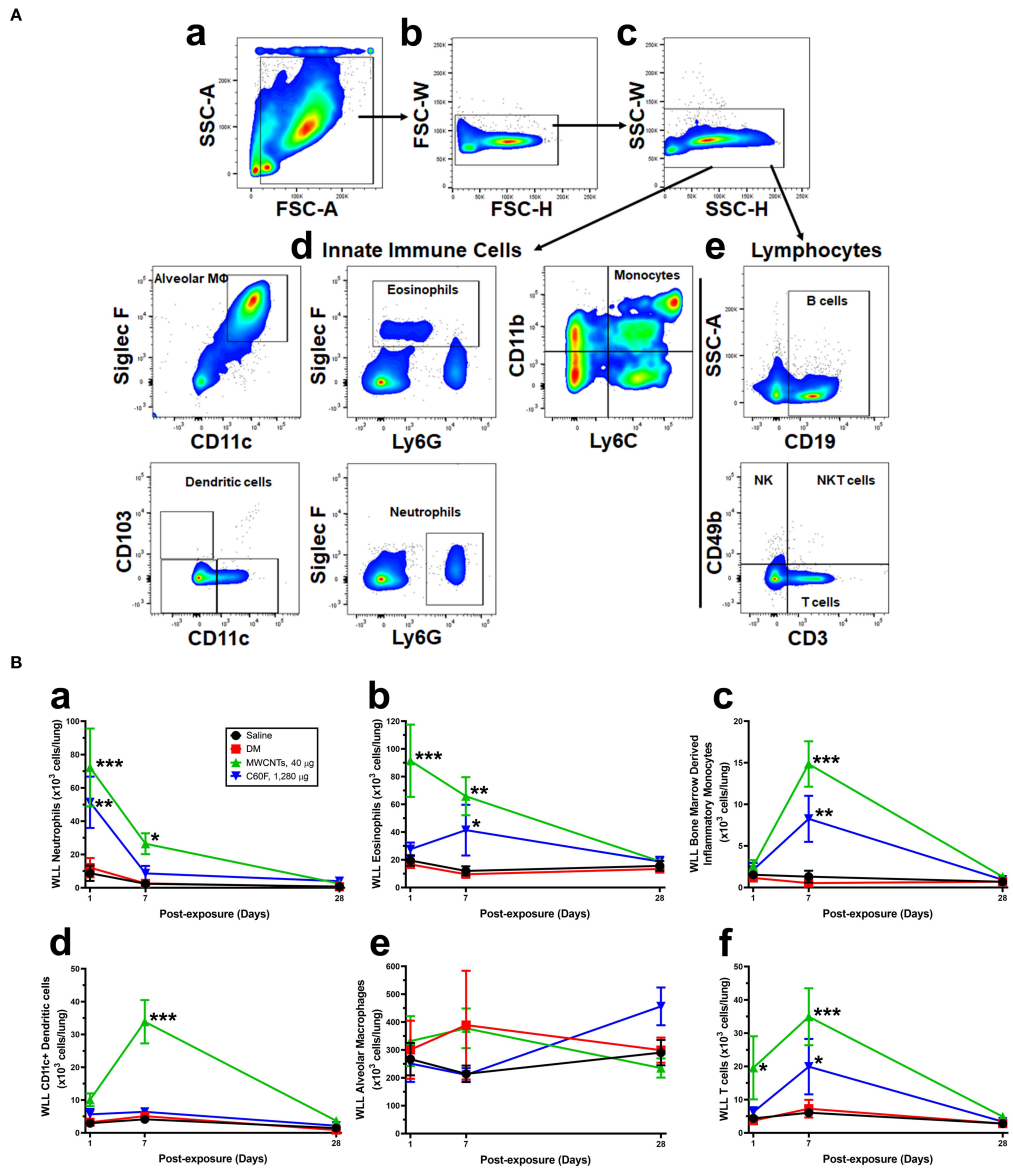
Flow cytometric profiling of immune cells in the mouse WLL fluid. Mice were treated as described in Figure 2 legend and were examined for immune cell profiling of the WLL fluid. **(A)** Gating strategies. Cells recovered from the mouse WLL fluid were resuspended in 1X PBS, stained with a fluorescent antibody cocktail, fixed, and then used to acquire cell populations using LSR II flow cytometer. Data were further analyzed using FlowJo software. Live cells were discriminated from dead cells, doublets, and debris first using FSC-A vs. SSC-A (a), then gating upon singlet populations in FSC-H vs. FSC-W view (b), then SSC-H vs. SSC-W view (c). Innate immune cells (d) and lymphocytes (e) were further separated. Gating panels were shown to subsequently identify different cell types based on the cell surface markers indicated on the X and Y axes of the flow cytometry pictographs. Cell populations were identified in order as follows: alveolar macrophages, eosinophils, neutrophils, lymphocytes, dendritic cells, and monocytes. **(B)** Quantification of immune cells. After cell type-specific gating using flow cytometry, neutrophils (a), eosinophils (b), bone marrow-derived inflammatory monocytes (c), CD11c+ DCs (d), AMs (e), and T cells (f) were calculated and total cell numbers (×10^3^ cells/lung) were expressed (*n* = 3). *p < 0.05; **p < 0.01; ***p < 0.001.

Specific cell types were identified based on cell surface markers and forward-side scatter profiles (Figure 5A). The numbers of WLL leukocytes recovered from saline- or DM-treated mice were similar to each other and remained unchanged over the time course (Figure 5B). MWCNTs induced a rapid increase in the number of neutrophils (Ly6G+ CD11b+) at day 1 post-exposure to a peak level at 72.20 × 10^3^ cells/lung, which is ~6.0-fold increase over DM control. The number decreased to 36.6% of the peak number at day 7, and to control level at day 28 (Figure 5Ba). C60F at 1,280 μg induced similar changes: at day 1, neutrophils were increased to a peak level at 51.33 × 10^3^ cells/lung, a 4.3-fold increase over DM control, and then declined to 16.8% at day 7, and to control level at day 28. Eosinophils (Ly6G+ Siglec F+) were also rapidly increased in MWCNT-exposed mice at day 1 to 91.46 × 10^3^ cells/lung, a 5.5-fold increase over DM control, which decreased to 65.6% at day 7 and to control level at day 28 (Figure 5Bb). On the other hand, in C60F-treated mice, eosinophils were only slightly increased which is not statistically significant at day 1 but were significantly increased to 41.39 × 10^3^ cells/lung at day 7, before returning to control level at day 28. The number of bone marrow-derived inflammatory monocytes (BMDIMs, Ly6C+ CD11b+) was not increased at day 1, but was drastically increased by 12.9-fold in MWCNT-treated mice and by 7.2-fold in C60F-treated mice at day 7, both of which declined to control level at day 28 (Figure 5Bc). The number of CD11c+ DCs was increased slightly at day 1 in MWCNT-exposed mice, but it was markedly increased to a peak level at 33.88 × 10^3^ cells/lung at day 7, a 3.4-fold increase over day 1 and 6.7-fold increase over DM control. The number of CD11c+ DCs declined to control level at day 28 (Figure 5Bd). In contrast, C60F exposure increased the number of CD11c+ DCs slightly at day 1 but did not further increase the number at day 7, and the number returned to control level at day 28. For alveolar macrophages (AMs, CD11c+ Siglec F+), there was no significant change in cell numbers of either MWCNT- or C60F-exposed mice in comparison to saline or DM control (Figure 5Be). The number of total T cells (CD3+ CD49b-) was also quantified (Figure 5Bf). In MWCNT-exposed mice, the number was significantly increased by 5.3-fold over DM control to 19.58 × 10^3^ cells/lung at day 1, and by 4.8-fold to 34.93 × 10^3^ cells/lung at day 7, before returning to control level at day 28. On the other hand, in C60F-exposed mice, the number was increased only slightly at day 1, but was increased significantly by 2.7-fold over DM control at day 7, before returning to control level. We also measured B cells (CD19+). Like T cells, the number of B cells increased by 3.6- and 2.9-fold over DM control at day 7 in MWCNT- or C60F-exposed mice, respectively, before returning to control level at day 28; however, at day 1, C60F, but not MWCNTs, increased the number of B cells significantly, suggesting an early effect of C60F on B cells, compared to MWCNTs (data not shown).

**Figure 6:**
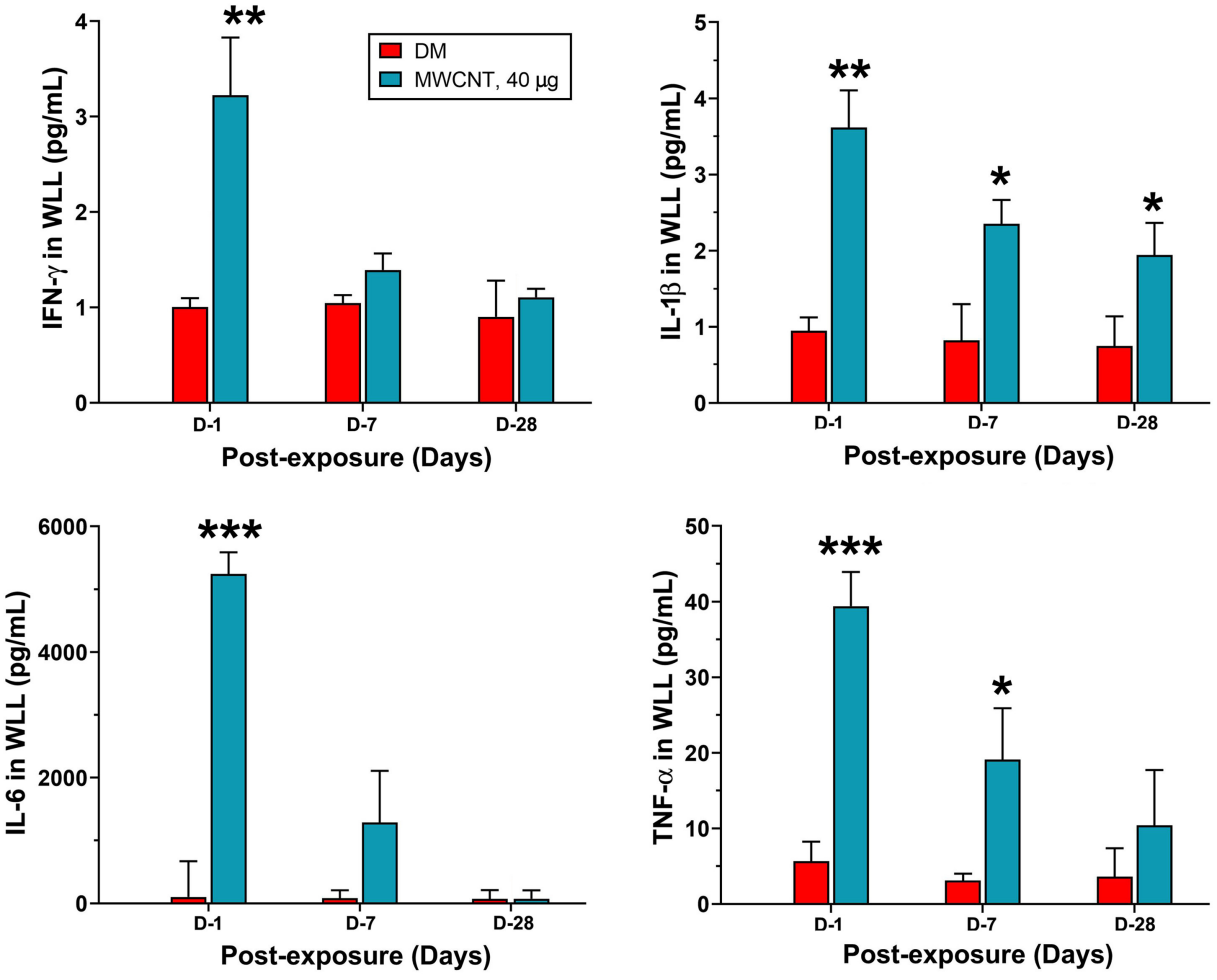
Quantification of proinflammatory cytokines in WLL fluids. Mice were treated as described in Figure 2 legend. At 1, 7, or 28 days post-exposure, mouse WLL fluids were prepared and used to measure IFN-γ, IL-1β, IL-6, and TNF-α levels by multiplex immunoassays. Data are expressed as mean ± SEM (*n* = 6). *p < 0.05; **p < 0.01; ***p < 0.001.

Acute inflammatory infiltration was corroborated with the rapid induction and secretion of proinflammatory cytokines in the WLL fluid. Figure 6 shows proinflammatory cytokine levels in WLL fluids from mice exposed to MWCNTs. IFN-γ was increased rapidly and markedly at day 1 by 3.2-fold over DM control, but the level decreased rapidly thereafter to nearly the basal level at day 28 post-exposure in the WLL fluid. IL-1β was increased to 3.55 pg/ml, a 3.9-fold increase over DM control. The level decreased to 66.2% at day 7 and to 54.9% at day 28. IL-6 was elevated to 5,250.34 pg/ml (52.7-fold increase), which decreased to 22.9% at day 7 and to basal level at day 28. Tumor necrosis factor (TNF) α was elevated to 38.9 pg/ml (7.8-fold increase) at day 1 over DM control, but the level rapidly decreased to 46.2% at day 7 and to 25.6% at day 28. Treatment with C60F at the 1,280 μg dose induced similar induction and subsequent decline of the cytokines in the lung (data not shown).

These findings indicate that MWCNTs at a low dose and C60F at a high dose induced a rapid increase followed by decline in neutrophilic infiltration and secretion of proinflammatory cytokines in WLL fluids. This result confirmed the pathological findings from lung tissue discussed above, which revealed a rapid initiation of acute inflammation followed by prolonged resolution of inflammation before progression to chronic lesions in mouse lungs exposed to MWCNTs or C60F. Besides neutrophils, MWCNTs and C60F also elicited dynamic changes of other immune cells in the lung that were similar between MWCNTs and C60F groups for BMDIMs, but differed substantially for eosinophils, CD11c+ DCs, and T and B cells (Figure 5B and data not shown). The significance of these differences in the induced infiltration of leukocytes by MWCNTs or C60F remains to be investigated.

### Temporal Elevation of Proinflammatory LMs and SPMs

**Figure 7:**
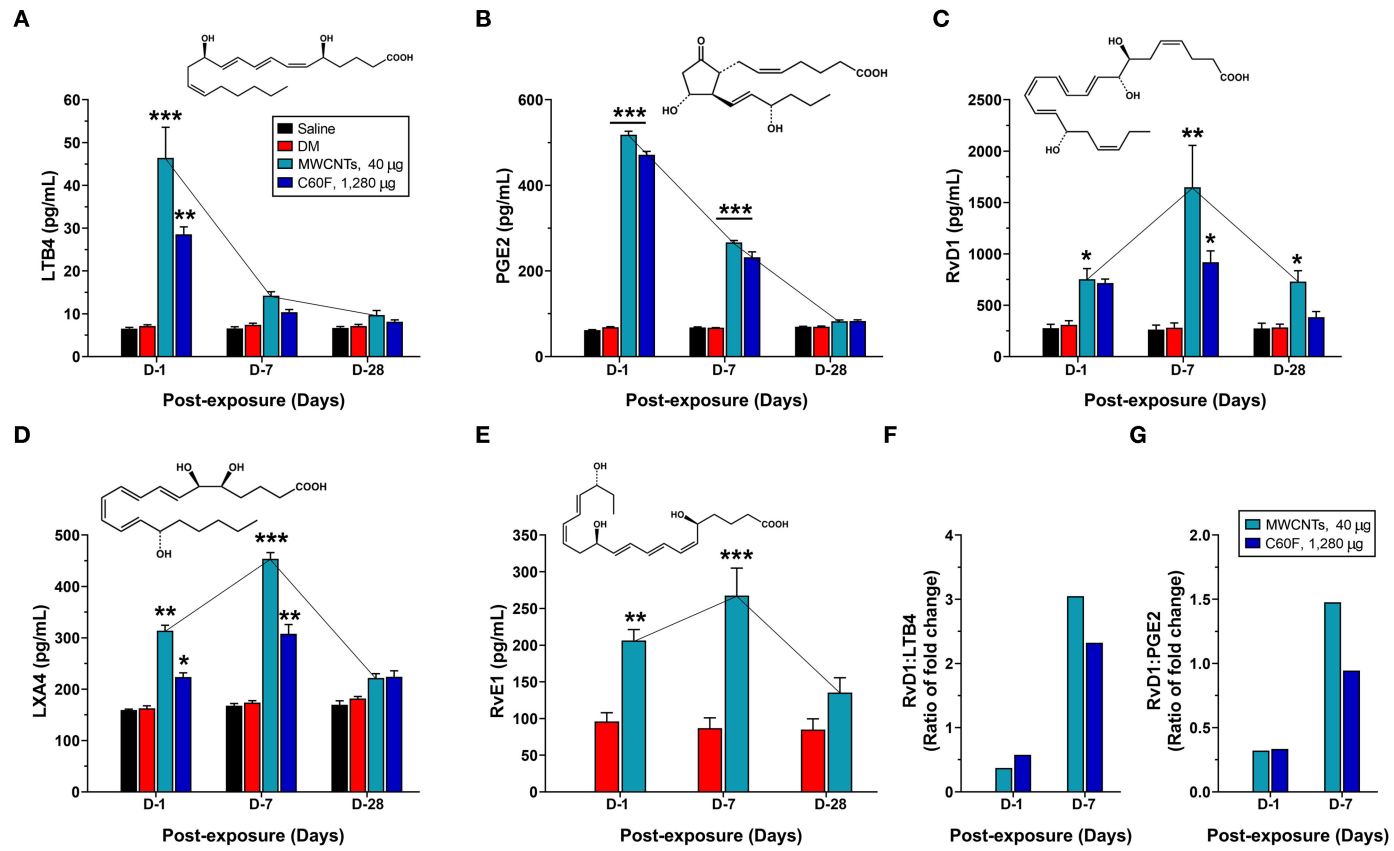
Quantitative measurement of pro-inflammatory LMs and SPMs in mouse WLL fluids. Mice were treated as described in Figure 2 legend and were examined for the levels of specialized LMs in the WLL fluid. Proinflammatory LMs LTB4 **(A)** and PGE2 **(B)**, or SPMs RvD1 **(C)**, LXA4 **(D)**, and RvE1 **(E)** were quantitatively measured using ELISA (*n* = 6). The ratio of fold change for RvD1/LTB4 **(F)** or RvD1/PGE2 **(G)** was further calculated for MWCNT- or C60F-treated samples at day 1 and day 7. *p < 0.05; **p < 0.01; ***p < 0.001.

The initiation and resolution of inflammation are tightly regulated by potent LMs (18, 26). Detection of specific types of secreted LMs is key to understanding the progression and resolution of inflammation. Therefore, we used well-established ELISA methods to measure the levels of proinflammatory LMs, including LTB4 and PGE2, and SPMs, including RvD1, LXA4, and RvE1, in WLL fluids from mice exposed to MWCNTs at 40 μg/mouse or C60F at 1,280 μg/mouse (Figure 7). Neither saline nor DM caused evident changes in the levels of the LMs. MWCNT treatment stimulated high levels of secretion of LTB4 at 46.4 ± 2.9 pg/ml, a nearly 6.5-fold increase over DM control, and PGE2 at 518.1 ± 8.4 pg/ml, a 7.6-fold increase over DM control, at day 1 post-exposure (Figures 7A,B). The LMs decreased drastically to 14.2 ± 0.4 pg/ml for LTB4 and to 266.4 ± 7.8 pg/ml for PGE2 at day 7, and further decreased to control levels at day 28. C60F induced a similar pattern of changes: both LTB4 and PGE2 reached their highest levels at day 1 post-exposure, i.e., 28.6 ± 1.7 pg/ml or a 4.0-fold increase over DM control for LTB4, and 471.6 ± 4.7 pg/ml or 6.9-fold increase over DM control for PGE2, which decreased to 10.4 ± 0.6 pg/ml for LTB4 and to 232.2 ± 12.6 pg/ml for PGE2 at day 7. Both returned to basal levels at day 28.

MWCNT treatment increased the level of RvD1 by 2.4-fold over DM control to 751.8 ± 105.6 pg/ml at day 1 post-exposure, which was further increased by 5.9-fold over DM control to a peak at 1648.2 ± 407.3 pg/ml at day 7, followed by reduction at day 28 to a level that was comparable to that of day 1 and was 2.6-fold higher than DM control (Figure 7C). C60F increased the level of RvD1 by 2.3-fold over DM control to 714.5 ± 39.6 pg/ml at day 1 post-exposure, which further increased by 3.9-fold over control to 918.333 ± 110.586 pg/ml at day 7, followed by decrease to control level at day 28. The amount of LXA4 was increased by MWCNTs at day 1 to 313.8 ± 10.7, a 1.9-fold increase over DM control, and was further increased at day 7 to a peak at 453.8 ± 12.3 pg/ml, a 2.6-fold increase over DM control, before returning to near control level (Figure 7D). C60F induced LXA4 at day 1 to 223.5 ± 8.2 pg/ml, a 1.4-fold increase over DM control, and at day 7 to 307.6 ± 18.3 pg/ml, a 1.8-fold increase over DM control, followed by reduction to near basal level. MWCNTs also induced RvE1 to 206.4 ± 14.8 pg/ml, a 2.2-fold increase over DM control, at day 1 post-exposure, and to a peak level at 267.4 ± 37.6 pg/ml, a 3.1-fold increase over DM control, at day 7, followed by reduction to near basal level (Figure 7E).

The ratios of fold changes for RvD1 vs. LTB4 and RvD1 vs. PGE2 at different exposure time are presented in Figures 7F,G, respectively. Both ratios were at low levels (~0.5) at day 1 in MWCNT-exposed lungs, but the ratios were increased to 3.1- and 1.5-fold, respectively, at day 7, which indicated a metabolic switch in the production of proinflammatory LMs to SPMs, as inflammation evolved toward resolution. C60F induced a similar pattern of changes in the ratios of RvD1 vs. LTB4 and RvD1 vs. PGE2. At day 28, the ratios remained larger than 1 for MWCNT treatment, and were near 1 for C60F treatment (data not shown). This MWCNT- or C60F-induced change in the ratio of fold change for RvD1 vs. LTB4 or PGE2 reflects a switch from rapid production of proinflammatory LMs to rapid production of SPMs as inflammation continues. This temporal elevation in the production of resolvins over LTs and PGEs would convert the local environment from being proinflammatory to pro-resolving and, thereby, promote the resolution of neutrophilic inflammation in mouse lungs.

### Progression From Type 1 to Type 2 Inflammation in Lung Tissue

**Figure 8:**
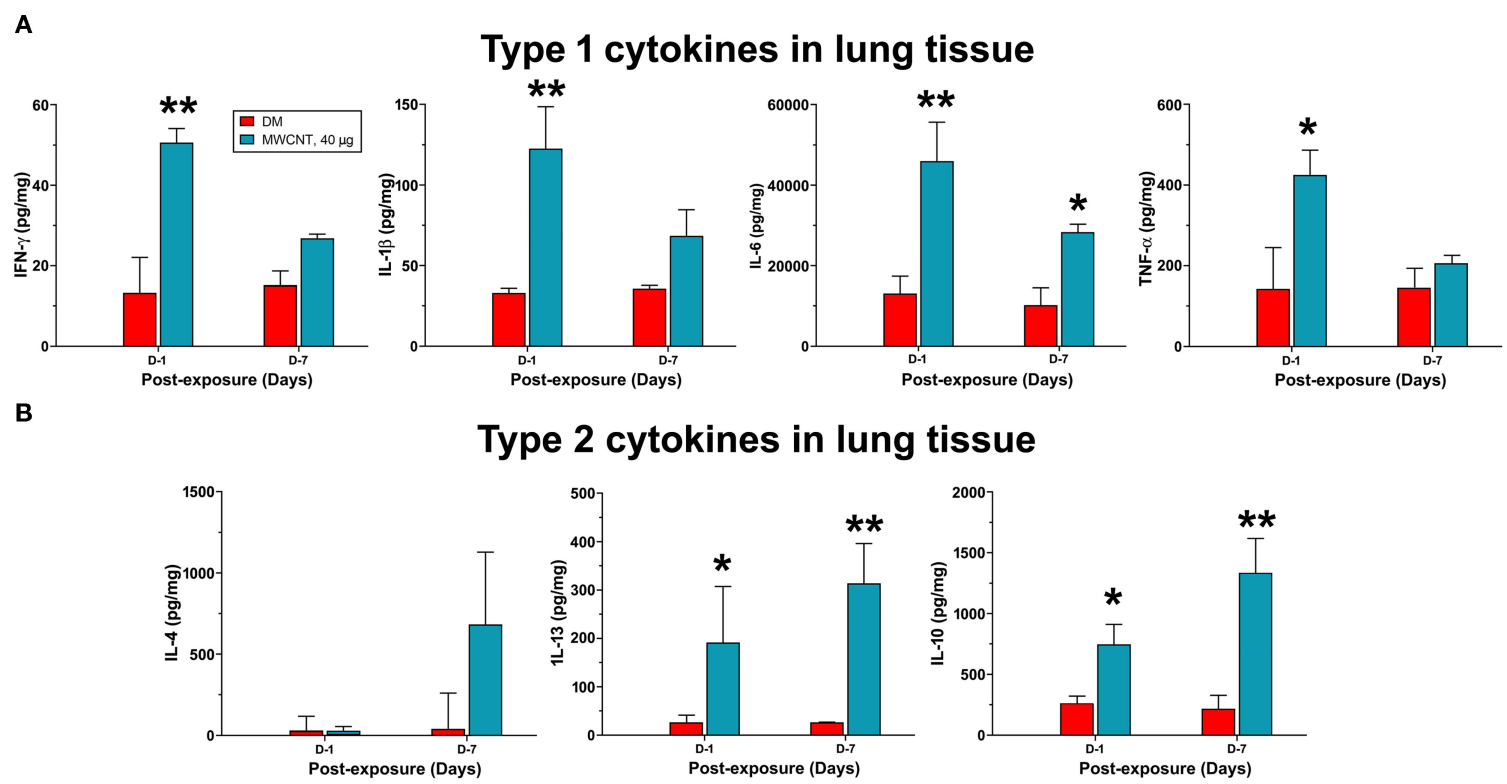
Quantification of type 1 and type 2 cytokines in lung tissue. Mice were treated as described in Figure 2 legend and the lung tissue extract was prepared. Twenty-five microliter of tissue extracts were used for multiplex immunoassays to determine the levels of type I cytokines IFN-γ, IL-1β, IL-6, and TNF-α **(A)**, or type II cytokines IL-4, IL-13, and IL-10 **(B)**. The concentration of each cytokine was presented as pg/mg protein after normalization with the amount of protein used and shown as mean ± SEM (*n* = 3). *p < 0.05, **p < 0.01.

The time-dependent resolution of neutrophilic inflammation and temporal elevation of SPMs in WLL fluids during resolution likely reflect alteration in the tissue response of lung parenchyma. Therefore, we examined the evolvement of inflammatory events that may promote the conversion of inflammation to resolution in lung tissue. We first compared the expression of proinflammatory and pro-resolving cytokines between days 1 and 7 post-exposure, during which time major resolution events were observed (Figure 8). Like cytokines in WLL fluids, proinflammatory cytokines IFN-γ, IL-1β, IL-6, and TNF-α were rapidly induced to high levels at day 1, with an increase of 3.9-, 4.7-, 3.6-, or 2.9-fold, respectively, compared with DM control (Figure 8A). Among the cytokines, IFN-γ is secreted by Th1 and ILC1 immune cells and is a major initiating cytokine of type 1 inflammation, whereas IL-1β, IL-6, and TNF-α are produced from effector cells, such as neutrophils and M1 macrophages, to mediate and amplify type 1 inflammatory responses in lung tissue. Therefore, this result supports the rapid induction and resolution of type 1 inflammation in the lung. Type 2 inflammation is characterized by the secretion of type 2 cytokines, typified by IL-4, IL-13, and IL-10. IL-4 and IL-13 are produced by Th2 and ILC2 cells that initiate type 2 inflammation, whereas IL-10 inhibits the polarization of Th1 cells, thereby inhibiting type 1 inflammation. As shown in Figure 8B, type 2 cytokines exhibited a contrasting pattern of expression from type 1 cytokines. IL-4 remained at a low level (48.7 pg/mg protein) at day 1 post-exposure but was elevated to a high level (741.2 pg/mg protein) at day 7 post-exposure. IL-13 was elevated at day 1 by 8.3-fold and was further increased by 13.7-fold at day 7 over DM control. Similarly, IL-10 was increased at day 1 by 3.1-fold and was further increased at day 7 post-exposure by 5.3-fold. Together, these findings indicate that, as type 1 inflammation is reduced in intensity, type 2 inflammation becomes predominant in the lung, which temporally correlated with the increased production of SPMs and resolution of neutrophilic inflammation in lung tissue.

**Figure 9:**
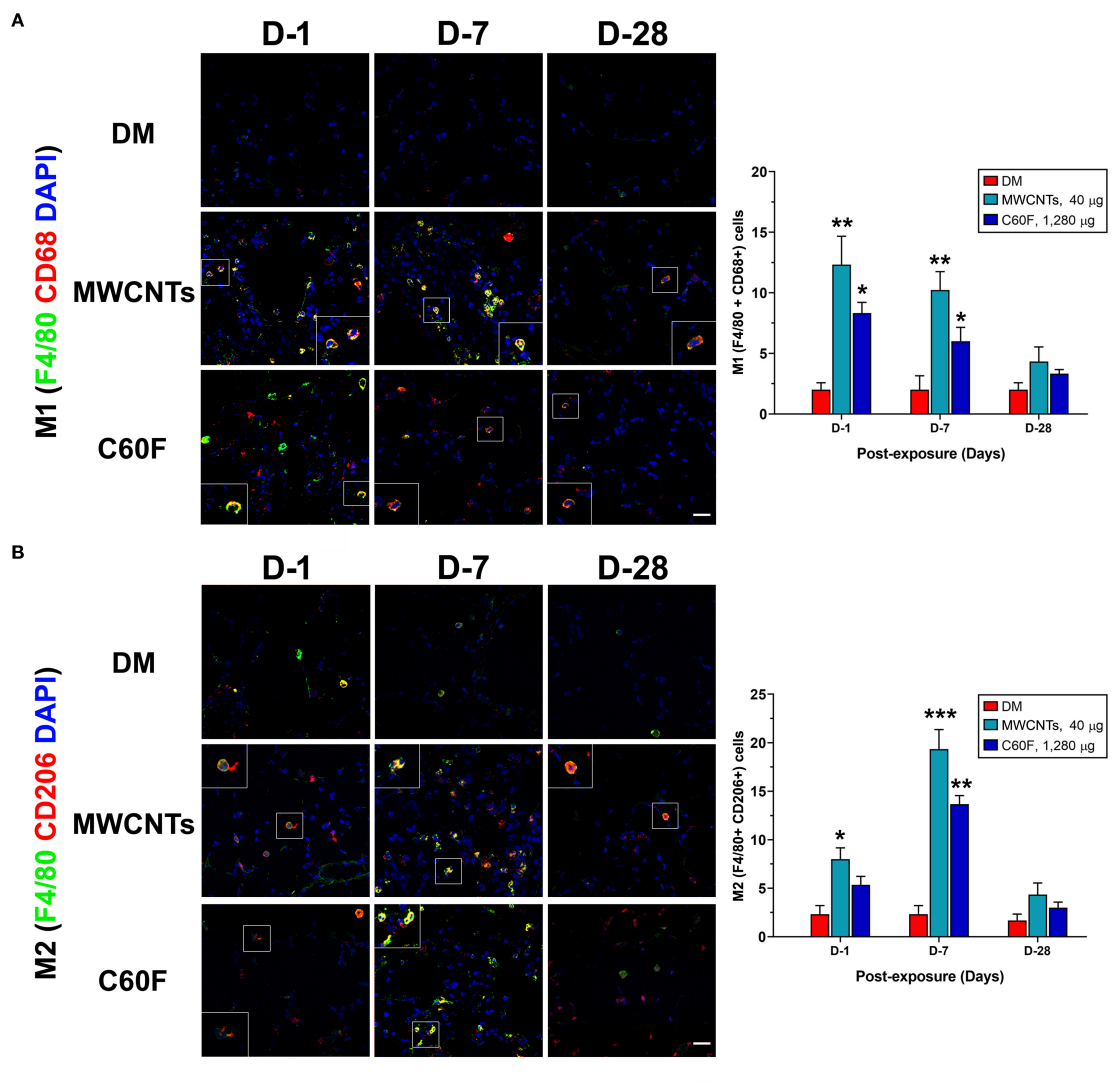
M1-M2 polarization in lungs exposed to MWCNTs or C60F. Mice were treated as described in Figure 2 legend. M1 or M2 polarization of macrophages was examined by double immunofluorescence staining with anti-F4/80 (green) and anti-CD68 (red) antibodies **(A)**, or anti-F4/80 (green) and anti-CD206 (red) antibodies **(B)** in lung tissue. Numbers of representative double positive cells per 100 cells were counted from captured images and shown as mean ± SEM (*n* = 3). Scale bar = 20 μm. *p < 0.05; **p < 0.01; ***p < 0.001.

In type 1 or type 2 inflammation, bone marrow-derived monocytes and tissue resident macrophages differentiate into M1 or M2 macrophages, respectively. Polarized M1 and M2 macrophages have distinct markers and metabolic and transcriptional profiles, and function as major effector cells. Moreover, polarized macrophages can be further separated into subgroups in an inducer-, time-, and context-dependent manner, though certain plasticity in the classification and function of polarized macrophages have been noted (37, 39). To examine whether the M1-M2 polarization takes place in MWCNT- or C60F-induced pulmonary inflammation and resolution, a set of markers for M1 or M2 macrophages were examined in lung tissue by double-label immunofluorescent staining of lung sections. In MWCNT-exposed lungs, the number of M1 macrophages (F4/80+ CD68+) was significantly increased, often in areas with MWCNT deposits, at all the time points examined in comparison to DM control. The highest number of positively stained cells was observed at day 1 with a 6.1-fold increase over control, which decreased slightly at day 7 and to near control level at day 28 (Figure 9A). C60F increased the number of M1 cells by 2.9-fold over DM control on day 1, which decreased at day 7 slightly and to near control level at day 28. Induction of M2 macrophages (F4/80+ CD206+) exhibited a different time course from that of M1 macrophages in that the number of M2 macrophages continuously increased from days 1 to 7 post-exposure, i.e., an increase by 3.9-fold over control at day 1 and by 9-fold over DM control at day 7 (Figure 9B). C60F-exposed lungs showed a similar pattern of increase in M2 cells, i.e., showing a 2.4-fold increase over DM control at day 1 and a 6.3-fold increase at day 7. At day 28, the level of M2 macrophages decreased to low levels that were slightly higher than DM control in both MWCNT- and C60F-treated mice. These results revealed that MWCNT or C60F exposure induced the differential enrichment of M1 and M2 macrophages in the lung that followed the progression from type 1 to type 2 inflammation in the lung. Moreover, the time-dependent reduction of M1 and increase of M2 macrophages correlated with the temporal reduction of proinflammatory LMs and elevation of SPMs during the resolution of inflammation. These findings implicate M1 and M2 in the differential production of the LMs during the initiation and resolution of inflammation, respectively.

### *In vivo* Induction of ALOX5AP in M1 and ALOX15 in M2 Macrophages by ENMs

**Figure 10:**
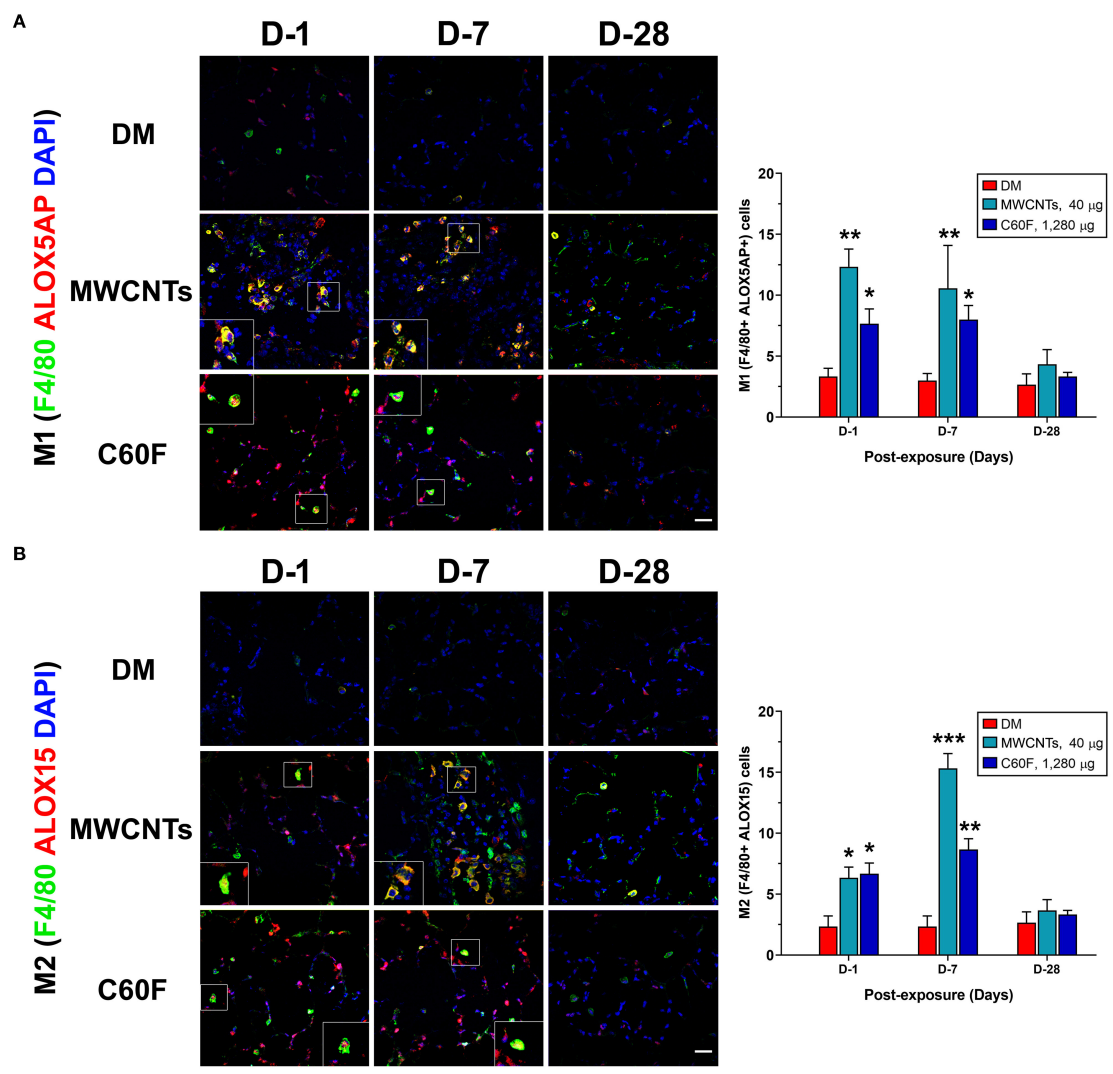
Macrophage polarization and lipid mediator pathway induction in mouse lungs exposed to MWCNTs or C60F. Mice were treated as described in Figure 2 legend and the lungs were examined. Macrophage polarization and lipid mediator pathway induction were examined by double immunofluorescence staining with anti-F4/80 (green) and anti-ALOX5AP (red) antibodies **(A)**, or anti-F4/80 (green) and anti-ALOX15 (red) antibodies **(B)**. Numbers of representative double positive cells per 100 cells were counted from captured images and shown as mean ± SEM (*n* = 3). Scale bar = 20 μm. *p < 0.05; **p < 0.01; ***p < 0.001.

Upon activation, M1 macrophages exhibit elevated expression of ALOX5AP (38). Activated ALOX5AP associates with ALOX5, leading to the activation of ALOX5, a rate-limiting step in the biosynthesis of LTB4 and PGE2 from endogenous AA. On the other hand, M2 macrophages express a high level of ALOX15 that is required for the synthesis of SPMs, i.e., the synthesis of LXs from AA and of RvDs, protectins, and maresins from DHA, during resolution of inflammation (27, 29). Therefore, we examined if the polarization of M1 and M2 macrophages by MWCNTs and C60F corresponds to preferential induction of ALOX5AP and ALOX15, respectively. The expression of ALOX5AP and ALOX15 proteins was examined in macrophages *in vivo* using double-label immunofluorescence staining (Figure 10). In MWCNT-exposed lungs, the number of ALOX5AP positive staining cells was induced markedly (F4/80+ ALOX5AP+) at day 1, a 3.9-fold over control, followed by a slight reduction at day 7, but nearly complete reduction to control numbers at day 28 (Figure 10A). C60F exposure significantly increased the number of F4/80+ ALOX5AP+ macrophages at day 1 (2.3-fold), which reduced slightly at day 7 and returned to control level at day 28. This pattern of changes in F4/80+ ALOX5AP+ macrophages paralleled to the polarization of M1 macrophages in the lung. The number of F4/80+ ALOX15+ cells was slightly increased with a statistical significance, compared to DM control, at day 1 in both MWCNT- and C60F-exposed lungs with 2.8- and 3.0-fold increases, respectively. The number was markedly elevated at day 7 in MWCNT-exposed lungs by 6.8-fold and in C60F-exposed lungs by 3.8-fold over DM control (Figure 10B). In both MWCNT- and C60F-exposed lungs, the numbers of F4/80+ ALOX15+ macrophages returned to near control level at day 28 post-exposure. This pattern of change in F4/80+ ALOX15+ macrophages paralleled to the polarization of M2 macrophages in the lung.

Together, these results indicate that M1 or M2 macrophages were differentially enriched and activated during MWCNT- or C60F-stimulated inflammation and resolution, with preferential induction of enzymes required for the synthesis of proinflammatory LMs in M1 macrophages or SPMs in M2 macrophages, respectively.

### Induced Synthesis of Proinflammatory LMs and SMs by ENMs in Polarized Macrophages

The observations from *in vivo* experiments discussed above suggest that polarization of M1 and M2 macrophages likely accounts for the time-dependent, differential production of proinflammatory LMs (LTs and PGE2) and SPMs (RvDs and LXs) in mouse lungs exposed to MWCNTs or C60F, respectively. We adopted an *in vitro* model of macrophage polarization to further investigate how the polarization of macrophages alters the synthesis of proinflammatory LMs and SPMs and whether ENMs affect this metabolic switch of LM production in polarized macrophages.

**Figure 11:**
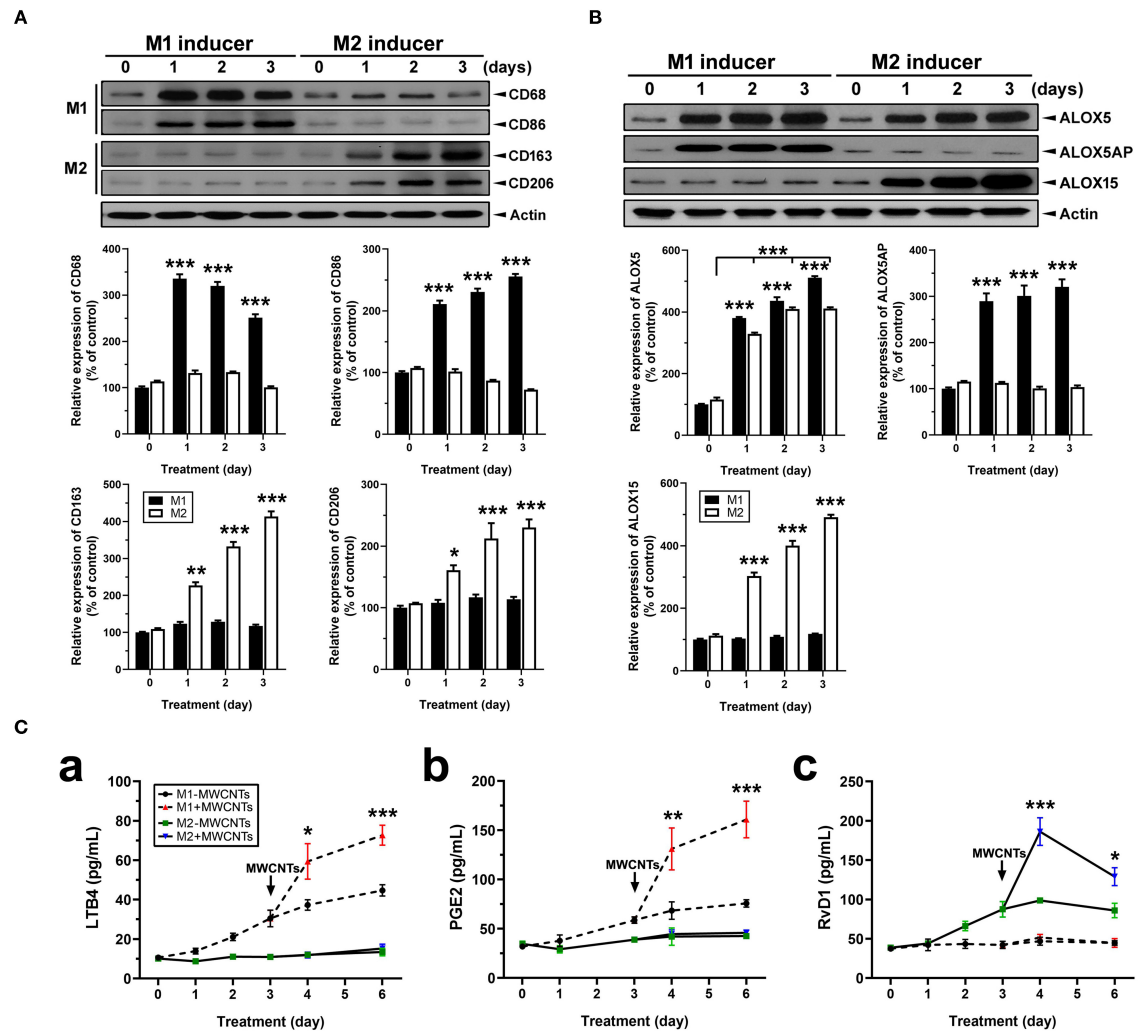
Macrophage polarization, lipid mediator pathway induction, and LM production. **(A)** Polarization of macrophages *in vitro*. Macrophage cells (J774A.1) were polarized by treating with M1 inducer (IFN-γ at 20 ng/ml plus LPS at 100 ng/ml) or M2 inducer (IL-4 at 20 ng/ml) for the indicated time. Cells were lysed and analyzed by Immunoblotting against M1 markers CD68 and CD86, M2 markers CD163 and CD206, or β-actin (loading control). Representative image was shown from 3 different experiments. The relative amount of each protein was expressed as % of vehicle control and quantification was shown as mean ± SEM. **(B)** Expression of ALOX pathway enzymes in M1 or M2 macrophages. M1 and M2 cells were analyzed by immunoblotting against ALOX5, ALOX5AP, ALOX15, or β-actin (loading control). Representative image was shown from 3 different experiments and quantification was shown as mean ± SEM (*n* = 3). **(C)** Effect of MWCNTs on LM production from M1 or M2 macrophages. Macrophages were polarized with M1 or M2 inducer for 3 days. Polarized cells were then treated with DM or MWCNTs (2.5 μg/ml) for 1, 2, or 3 days. The cell-free culture media were collected for quantification of LTB4 (a), PGE2 (b), or RvD1 (c) using ELISA (*n* = 6). *p < 0.05; **p < 0.01; ***p < 0.001.

Macrophages (J774A.1) were differentially polarized for 1, 2, and 3 days. Polarization was confirmed by detection of the expression of M1- or M2-specific marker proteins using immunoblotting of the marker proteins and quantification of the protein bands (Figure 11). Expression of CD68 and CD86, two surface markers for the M1 phenotype, was evident at day 1 and persisted to day 3 in M1 inducer-treated, but not M2 inducer-treated, cells (Figure 11A). Expression of CD163 and CD206, two surface markers for the M2 phenotype, was induced at day 1 and continued to increase through day 3 in M2 inducer-treated, but not M1 inducer-treated, cells (Figure 11A). After differential polarization of macrophages, expression of enzymes involved in SPM biosynthesis was examined by immunoblotting. Induced expression of ALOX5 was consistently observed at each time point in both M1 and M2 macrophages, indicating it was induced by both M1 and M2 inducers. ALOX5AP was evidently induced in M1, but not M2, macrophages. On the contrary, the ALOX15 protein was strongly and continuously induced in M2, but not M1, macrophages (Figure 11B).

We then examined the temporal biosynthesis of proinflammatory LMs or SPMs in M1 or M2 macrophages and how MWCNTs affect this process. Macrophages were polarized with M1 inducers or M2 inducers for 3 days, respectively. Polarized macrophages were then incubated with DM or MWCNTs (2.5 μg/ml) for additional 1, 2, or 3 days. The cell-free culture media were assayed for production of LTB4, PGE2, and RvD1 by ELISA. LTB4 was produced throughout the polarization with a steady increase by M1, but not M2, macrophages. MWCNTs highly elevated the LTB4 production by M1, but not M2, cells (Figure 11Ca). A similar pattern was observed for PGE2 production by M1, but not M2, cells (Figure 11Cb). On the other hand, RvD1 was apparently produced by M2, but not M1, macrophages upon polarization. MWCNTs markedly increased the RvD1 production by M2 cells, with a peak at day 1, whereas no apparent increase in the RvD1 level was observed in the culture media of M1 macrophages (Figure 11Cc). These findings indicate that MWCNTs differentially augment the biosynthesis of proinflammatory LMs or SPMs in M1 or M2 macrophages, respectively, by way of induction and activation of specific ALOX pathways responsible for the synthesis of corresponding LMs from endogenous substrates.

## Discussion

**Figure 12:**
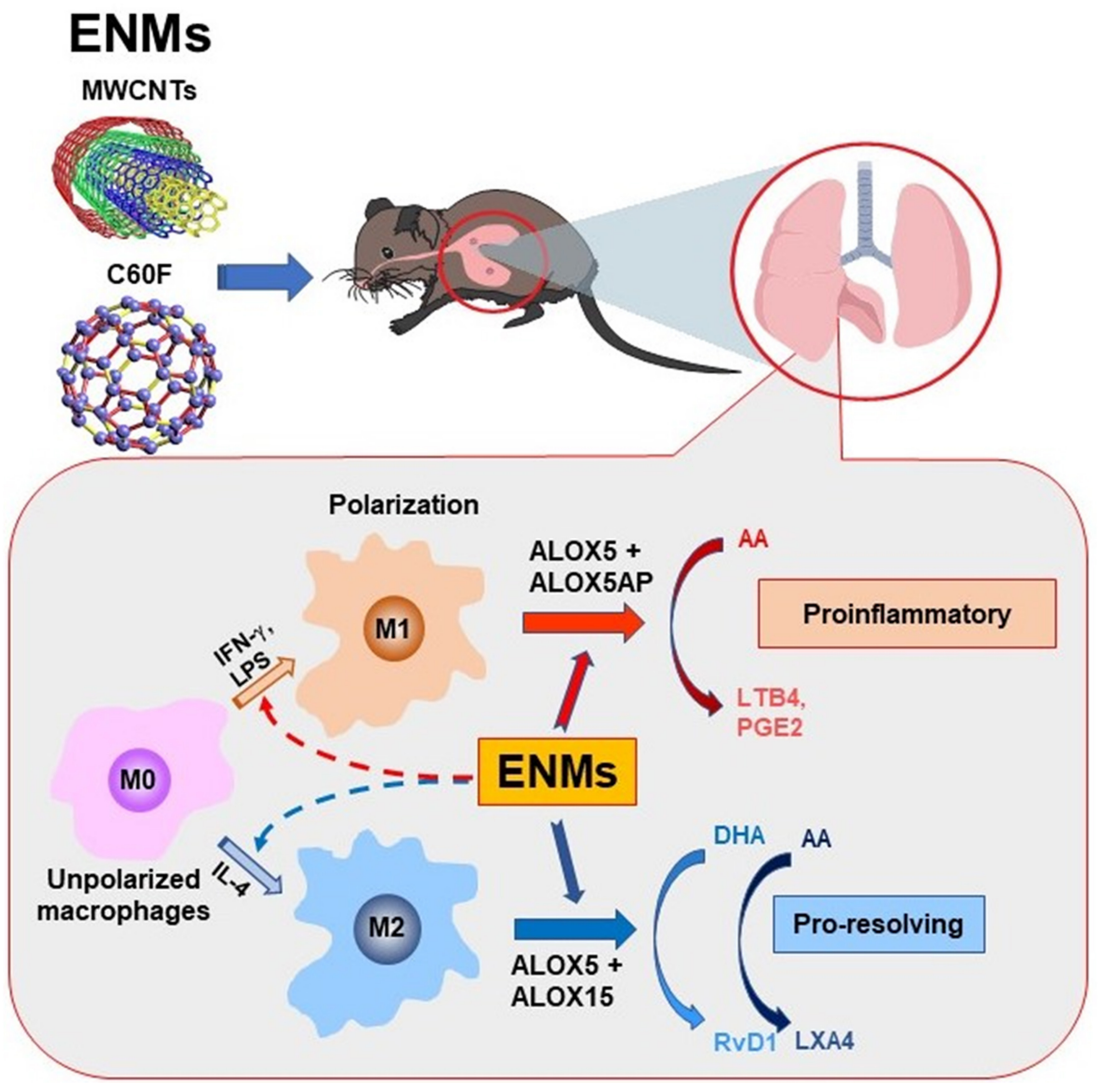
Induced biosynthesis of specialized LMs through M1- and M2-associated pathways. Polarization of M1 and M2 macrophages alters their ALOX pathways for the biosynthesis of proinflammatory LMs or SPMs and, thereby, regulate the initiation and resolution of neutrophilic inflammation in a time-dependent manner. In this model, exposure to inflammatogenic and fibrogenic ENMs, such as fiber-like MWCNTs, induces the production and secretion of LTB4, PGE2, and other proinflammatory LMs from M1 macrophages via induced expression of ALOX5 and ALOX5AP, which stimulates the rapid initiation of neutrophil infiltration and proinflammatory cytokine secretion. The continued presence of ENMs in the lung stimulates the production of RvD1 and other SPMs from M2 macrophages by inducing ALOX5 and ALOX15, which converts the lung environment from being proinflammatory to pro-resolving, thereby promoting the resolution of neutrophilic inflammation.

Inflammation is a common, early response to exposure to ENMs like CNTs in the lung. If unresolved, pulmonary inflammation may evolve into chronic and progressive disease conditions, including fibrosis and malignancy (5, 7, 9, 35, 42). In these scenarios, acute inflammation is immediately followed by resolution events. However, pulmonary clearance of inhaled particles and nanoparticles is often inefficient, and deposits of particulates may persist in the lung parenchyma and, in the case of asbestos fibers and certain MWCNTs, the pleural space, for a long period of time. As a result, resolution of inflammation induced by inhaled particulates is often incomplete and prolonged, which stimulates the development of granulomatous inflammation, fibrosis, lung cancer, and pleural plague and mesothelioma (35, 42). Although the inflammatory and fibrotic effects of CNTs and some other ENMs have been well documented (3, 4, 11, 52), how CNT- and other ENM-induced pulmonary inflammation is resolved remains unstudied. In this report, we show that fiber-like and fibrogenic MWCNTs, i.e., Mitsui-7, at a low dose, and spherical, low toxicity fullerenes, i.e., C60F, at a high dose, induced the cellular and molecular events for the resolution of pulmonary acute inflammation, characterized by reduction of neutrophils, polarization of M1 and M2 macrophages, and phenotype-specific production of specialized LMs. At the molecular level, pulmonary exposure to the ENMs induced the production and secretion of proinflammatory LMs, including LTB4 and PGE2, from M1 macrophages via induction of ALOX5 and ALOXAP. The continued presence of ENMs stimulated the polarization of M2 macrophages and the production of SPMs, including RvD1, RvE1, and LXA4, through induction of ALOX5 and ALOX15. This time-dependent switch in the polarization of macrophages and activation of ALOX pathways converted the microenvironment in the inflamed lung from being proinflammatory to pro-resolving, thereby promoting the resolution of neutrophilic inflammation (Figure 12).

Acute inflammation is typically characterized by increased blood flow, capillary expansion, leukocyte infiltration, and enhanced production of chemical mediators (16). The presence of neutrophils at inflamed tissue is a hallmark of acute inflammation. This rapid and heightened influx of neutrophils helps eliminate inhaled microbial and non-microbial insults but, at the same time, causes damage to lung tissue and is thus considered unfavorable to tissue homeostasis (32). Accordingly, a key step in the resolution of inflammation is the reduction of neutrophils in inflamed tissue, which is accomplished by reducing the recruitment of neutrophils and inducing the programmed death, such as apoptosis, of leukocytes, followed by removal of dead cells via active phagocytosis known as efferocytosis (32, 53). Our flow cytometry profiling of WLL fluid cells revealed that the number of WLL neutrophils is the highest at day 1 in the early response to low dose MWCNTs or high dose C60F (Figure 5), which was consistent with histopathological findings (Figure 2). Accumulation of neutrophils in lung tissue was associated with deposits of ENMs. Additionally, proinflammatory cytokines IFN-γ, IL-1β, IL-6, and TNF-α in both the WLL fluid and lung tissue were rapidly increased to peak levels at day 1 post-exposure (Figures 6, 8). Therefore, both ENMs stimulated acute and prominent neutrophilic inflammation. WLL fluid neutrophils declined to 36.6% of the peak number for MWCNT- and 16.8% for C60F-treated mice at day 7 post-exposure and further decreased to control levels at day 28 in both groups. The levels of IFN-γ, IL-1β, IL-6, and TNF-α in WLL fluids and lung tissue were also rapidly reduced in parallel to neutrophils. Histopathology results revealed the resolution of acute neutrophilic inflammation at day 7, with a concurrent shift to granulomatous inflammation following MWCNT exposure. Together, these findings revealed a rapid initiation phase and an extended resolution phase of the inflammatory response to low dose MWCNTs or high dose C60F exposure.

Macrophages play a central role in resolution by orchestrating acute inflammation toward recovery. Macrophages are actively involved in the clearance of pathogenic microorganisms and non-microbial particles, including mineral particulates and ENMs, and the efferocytosis of leukocytes and damaged tissue during pulmonary inflammation (33–35). Upon activation by microbial or sterile stimuli, macrophages polarize to subgroups that exhibit distinctive phenotypes and perform specific functions in an inducer-, time-, and context-dependent manner, though certain plasticity in the subtypes and functions of polarized macrophages has been described (37, 39). Of note, polarization of M1 and M2 macrophages often occurs during the development of type 1 or type 2 inflammation, respectively, and the polarization of macrophages to M2 subtypes during type 2 inflammation has been implicated mechanistically for the development of fibrosis induced by a variety of fibrogenic signals including ENMs (13, 36, 54). Therefore, analysis of macrophage polarization upon ENM exposure could provide a novel cellular and molecular link at the interphase between pulmonary acute inflammation and resolution progression.

We have previously shown that MWCNTs elicit apparent activation and reprogramming of macrophages in the lung *in vivo* through specific signaling pathways (41). Polarization of M1 and M2 macrophages was characterized in details by using a combination of markers, including (a) cell surface markers, i.e., CD86 and MHC II for M1 and CD206 and CD163 for M2; (b) metabolic and functional markers, i.e., iNOS for M1 and ARG1, FIZZ1, and YM1 for M2; and (c) activation of key transcriptional pathways, i.e., activation of STAT1 and IRF5 signaling for M1 and of the STAT6/STAT3 and IRF4 pathway for M2, at protein and cellular levels in mouse lungs. In the current study, induced polarization of macrophages was characterized for specific and dynamic time courses and identified by surface marker expression during the initiation and resolution phases of lung inflammation induced by MWCNTs or C60F in mouse lungs *in vivo* (Figure 9). Together, these data consistently and clearly demonstrated that M1 macrophages expressing signature M1 markers were increased at day 1 rapidly and markedly, but the number of M1 cells decreased substantially at day 7 post-exposure and continued to decrease thereafter. On the other hand, M2 macrophages expressing signature M2 markers were increased in numbers at day 1 and continued to increase to reach a peak level at day 7 post-exposure to either MWCNTs or C60F, demonstrating a contrasting time course to that of M1. Furthermore, polarization of M1 macrophages correlated with the elevation of type 1 proinflammatory cytokines IFN-γ, IL-1β, IL-6, and TNF-α in lung tissue with peak levels at day 1, which indicates the development of acute type 1 inflammation, whereas polarization of M2 macrophages correlated with the increase of type 2 cytokines IL-4, IL13, and IL-10 with peak levels at day 7, which indicates the evolvement of type 2 inflammation (Figure 8). This differential and time-dependent activation of M1 and M2 polarization and elevation of type 1 and type 2 inflammation cytokines have implications for the development and resolution of lung inflammation induced by particles and ENMs. In this respect, M1 macrophages and type 1 cytokines promote acute inflammation by stimulating the recruitment of neutrophils and other inflammatory leukocytes, and increasing the production and release of microbe-killing agents to cause tissue damage, whereas M2 macrophages and type 2 cytokines are enriched after M1 induction and are positioned to inhibit M1-elicited type 1 inflammation and tissue damage and, at the same time, promote tissue debridement and repair. This delayed activation and polarization of M2 macrophages and elevation of type 2 cytokines correlate well with the decline of acute inflammation and subsequent transition to the resolution phase of inflammation in mouse lungs *in vivo*. The mechanism by which MWNTs stimulate M1 and M2 polarization in the lung remains to be elucidated. MWCNTs have not been shown to induce the full-scale polarization of cultured macrophages to M1 or M2 cells *in vitro*. It is possible that a permissive microenvironment, such as the co-existence of other cell types, including epithelial and endothelial cells and PMNs, is needed for macrophage polarization.

Resolution of inflammation is highly coordinated and specifically regulated by a variety of mediators (18). Defining endogenous mediators of resolution could help understand the mechanisms by which effector cells, including monocytes and macrophages, are involved in the resolution of inflammation induced by MWCNT exposure. M1 macrophages are activated earlier than M2 macrophages upon exposure. This result with macrophages is consistent with the spatial and temporal formation of distinct LMs obtained *in vivo* from mice exposed to microbes, wherein proinflammatory or pro-resolving LMs were synthesized during inflammation initiation, such as LTB4 and PGE2, or during resolution of inflammation, i.e., SPMs, such as RvDs, RvEs, and LXA4 (55, 56). In this study, we observed time-dependent elevation of proinflammatory LMs at day 1 and of pro-resolving LMs at the later stage, with a marked increase in the ratio of fold change for RvD1 vs. LTB4 or PGE2 at day 7 post-exposure. This change reflects a metabolic switch from rapid production of proinflammatory LMs to rapid production of SPMs, as the resolution of inflammation evolves and polarization of macrophages converts from M1-predominating to M2-predominating (Figures 7–9). This M1- and M2-dependent production of proinflammatory or pro-resolving LMs *in vivo* was further corroborated with findings from polarized macrophages *in vitro*, which showed that MWCNT treatment induced LTB4 and PGE2 production in M1 macrophages that expressed high levels of CD68 and CD86 markers, but not in M2 macrophages that expressed high levels of CD163 and CD206 markers (Figure 11). On the other hand, MWCNTs increased RvD1 in M2, but not M1, macrophages. Together, these *in vivo* and *in vitro* data support that the divergent temporal and spatial polarization of M1 and M2 macrophages evokes proinflammatory or pro-resolving phenotypic LM profiles after exposure and, thereby, promote the progression from inflammation to resolution.

These distinct LM signal profiles might determine different functions of M1 macrophages, mostly proinflammatory, vs. M2 macrophages, mostly pro-resolving, phenotypes (57). During polarization of macrophages, M1 and M2 macrophages acquire the sequential expression of heterogeneous LM pathways. COX1 and COX2 are major enzymes to catalyze the formation of PGs from AA and ALOX5 mediates the formation of LTs from AA, whereas ALOX15 and ALOX5 catalyze the production of LXs from AA and RvDs from DHA (28). Of note, COX2 can be acetylated by aspirin and S-nitrosylated in the presence of statins. Acetylation or S-nitrosylation at the active site of COX2 converts COX2 from being PG-producing to SPM-producing, which leads to the transcellular production of aspirin-triggered (AT) SPMs, including 15-epi-lipoxins, AT-RvDs, AT-RvEs, and resolving Ts (RvTs) (58, 59). While COX1 is constitutively expressed, COX2 and its products were induced by ENMs in cultured macrophages and in mouse lungs (60, 61). In the current study, we found that the ALOX5 pathway was induced, predominantly in M1 macrophages, both *in vivo* and *in vitro* (Figures 10A, 11B). Therefore, the inflammation-initiating COX-2- and ALOX5-mediated pathways are rapidly induced and predominated in M1 macrophages. Our study also identified that MWCNTs induced an abundant production of bioactive SPMs from endogenous substrates via ALOX15 (15-LOX in humans) in M2 macrophages and the ALOX15 enzyme was induced by ENMs in M2 cells both *in vivo* and *in vitro* (Figures 10B, 11B).

Both M1 and M2 cells express the ALOX5 protein, but M1 macrophages expressed more ALOX5AP than M2 cells, as confirmed in M1 inducer-treated cells (Figures 10A, 11B). Translocation of ALOX5 and its subsequent interaction with ALOX5AP determines M1-specific LM generation (38). ALOX15 is essentially absent in M1 macrophages, and thus RvD1 formation is very low, just above the limit of detection, in M1 cells (Figure 11). In contrast, the pro-resolving M2 macrophages express a high level of ALOX15 that preferably produces SPMs (Figures 10B, 11B). A strikingly higher level of ALOX15 protein expression in M2 than M1 cells accounts for the greater production of SPMs by M2 cells, whereas a higher level of ALOX5AP may support superior LTB4 and PGE2 formation in M1 than M2 macrophages. These findings are consistent with the well-appreciated proinflammatory role of M1 macrophages and the function of M2 in helping orchestrate the resolution of inflammation and tissue repair (34, 57). Whether the production of AT-SPMs occurs during, and is functionally associated with, the polarization of M1 and M2 macrophages remain to be investigated.

ALOX-mediated LM-SPM production is highly dependent on the translocation of ALOX5 and ALOX15 to where those enzymes meet specific endogenous substrates and convert downstream intermediates. The translocation and full enzymatic activities of ALOX5 and ALOX15 require intracellular Ca^2+^ signaling (62, 63). MWCNTs-treated cells have been shown to have elevated intracellular Ca^2+^ levels via membrane calcium channel activation (64). Additional studies are needed to identify intracellular Ca^2+^ signaling activation by different types of ENMs for activation of the ALOX pathways in M1 and M2 macrophages, respectively. Upon activation, differential intermediate precursors and LMs can be generated in a series of enzymatic reactions. Our results showed that representative LMs like LTB4 and PGE2 from M1 or RvD1 and LXA4 from M2 macrophages were generated and were detected after type-specific activation. However, the inflammatory system is complex and proinflammatory LMs and SPMs are diverse. A pro-resolving cascade becomes active during resolution, whereby one SPM could induce another one. Further investigation using liquid chromatography with tandem mass spectrometry (LC-MS-MS) will be needed for identification of other types of LMs like proinflammatory PGD2 or pro-resolving RvD5, RvE3, and maresin 1, and precursors like 5-hydroxyeicosatetraenoic acid or 17-hydroxy-docosahexaenoic acid increased by MWCNT exposure. This is crucial to defining intracellular pathways and targets that could resolve inflammation, especially, in the context of chronic inflammatory disease like lung fibrosis.

Inflammation and its resolution involve the functions of diverse innate and adaptive immune cells in inducer-, time-, and context-dependent manners. In addition to neutrophils and monocytes/macrophages, exposure to ENMs induced dynamic changes of other types of immune cells, some of which appear to be ENM-specific, as demonstrated by cell profiling of WLL fluid cells after ENM exposure (Figure 5). It is known that insufficient resolution of inflammation contributes to chronic pulmonary disease and lung fibrosis (25). Moreover, chronic inflammation is implicated in pulmonary fibrosis pathogenesis and is associated with persistent activation of immune responses that are largely controlled by DCs (65). This notion is supported by the observation that large numbers of DCs infiltrate into the lungs of patients with idiopathic pulmonary fibrosis (66). Our flow cytometric results revealed that DCs in WLL fluids in response to MWCNTs, but not C60F, were dramatically increased at day 7 post-exposure when granulomatous inflammation is characterizing. DCs capture antigens, migrate to draining lymph nodes and lung tissue, and present antigen to T cells. Our flow cytometric results also showed that the number of T cells was significantly increased in WLL fluids in response to MWCNTs at day 1 and day 7, and to C60F at day 7, post-exposure. Notably, the overall pattern of increase in DCs and T cells accorded well with those of M2 macrophages and type 2 inflammation. These results support the notion that the association of DCs with antigen-experienced T cells may drive an efficient effector immune response to initiate pulmonary fibrosis within ENM-treated lungs. The functions and mode of action of DCs in the resolution of inflammation induced by ENMs are not clear at the present time. DCs constitute an essential interface between innate sensing of pathogens and activation of adaptive immunity that involves a wide range of mechanisms and responses by different DC subsets (67). The mechanisms by which DC's phenotypic maturation, accumulation in lung tissue, and expression and interaction with other lung DC subsets are controlled need to be further investigated in future studies, which helps better understand the early inflammatory responses to different type of ENMs, in particular, during resolution of inflammation.

Eosinophils are typically associated with parasitic infection and allergic responses, such as asthma. Recent findings reveal critical roles of eosinophils in the initiation of type 2 immune responses (13, 54). Upon ENM exposure, deposition of ENMs in lung tissue causes damage to airway and alveolar epithelial cells, which release alarmins, including IL-33, IL-25, and thymic stromal lymphopoietin (TSLP) locally (68). These alarmins recruit innate immune cells, such as eosinophils, to provide the initial production of IL-4, which strongly stimulates the initiation and amplification of type 2 immune responses, characterized by the polarization of T helper (Th) 2 cells and M2 macrophages (40) and secretion of type 2 cytokines (69). Secretion of IL-5 further stimulates the recruitment of eosinophils (61). Our data revealed that MWCNTs stimulated a rapid increase in the number of WLL fluid eosinophils with a peak at day 1 post-exposure (Figure 5). This result supports an early function of eosinophils in the initiation of immune responses to pulmonary exposure to MWCNTs. Exposure to C60F at a high dose increased the number of eosinophils at day 7, but not day 1, post-exposure, which may contribute to a weaker type 2 immune response to C60F than MWCNTs and, accordingly, reduced chronic inflammatory and fibrotic responses in C60F-exposed lungs, as compared to MWCNT-exposed lungs.

This study demonstrates that the M1- and M2-mediated responses were markedly induced in the early phase response in the lung in an ENM- and time-dependent manner. The differential activation of M1- and M2-dependent ALOX pathways mediates the induced synthesis of proinflammatory or pro-resolving LMs from endogenous substrates and, thereby, coordinates the prolonged resolution of pulmonary inflammation in the presence of persistent ENMs (Figure 12). Macrophage polarization-dependent activation of ALOX pathways in mouse lungs described above has been observed with human peripheral blood monocytes challenged by microbial pathogens (38). Given the critical roles of proinflammatory LMs and SPMs in the initiation, amplification, and resolution of inflammation, knowledge obtained from these pathways has translational potentials. For instance, the pathways can serve as new targets for therapeutic targeting, preventive intervention, and biomarker-based risk assessment for human disease caused by insults that include, but not limited to, fibrogenic and tumorigenic inhaled particles, fibers, and ENMs. In this connection, it has been shown that dietary supplement of ω-3 polyunsaturated fatty acids, such as DHA, prevented silica-induced inflammatory and autoimmune responses *in vitro* and in susceptible animal models (70–72). Pulmonary exposure to ozone caused lung inflammation associated with reduced SPM production; conversely, pretreatment with DHA reduced ozone-caused lung inflammation (73). Certain SPMs have demonstrated efficacy in treating inflammatory diseases caused by infection or sterile insults such as tobacco smoke (18, 56, 74).

Humans encounter numerous micro- and nano-sized particulates through inhaled air from environmental, occupational, and commercial sources on daily basis. These particulates differ substantially in their physicochemical properties and toxicity but would all cause lung disease if the amount and duration of exposure are sufficiently high, best exemplified by silicosis and asbestosis caused by highly toxic mineral particles, i.e., silica, and fibers, i.e., asbestos, and black lung disease caused by low toxicity coal dust (75, 76). The rapid development and utilization of ENMs raised new concerns for possible adverse effects on exposed populations. This study demonstrates that MWCNTs at a low dose and C60F at a high dose stimulate the rapid initiation of acute inflammation, followed by extended resolution of inflammation in mouse lungs through the dynamic recruitment and activation of immune cells—in particular, the polarization of macrophages. Moreover, the lesions in MWCNT-exposed mice progressed to fibrotic granulomas, whereas it remained as alveolar histiocytosis in C60F-exposed mice. The large difference in potency and disease outcomes between MWCNTs and C60F found in this study provides a unique opportunity for further investigation of how distinct particulates with diverse physicochemical properties cause pulmonary inflammation and how the resolution or a “failed” resolution of inflammation contributes to the pathogenesis of progressive and lethal outcomes such as organ fibrosis and cancer in the lung and the pleura in humans.

## Data Availability Statement

All datasets generated for this study are included in the article/supplementary material.

## Ethics Statement

The animal study was reviewed and approved by the Institutional Animal Care and Use Committee at the National Institute for Occupational Safety and Health, Morgantown, WV, USA.

## Author Contributions

QM, DB, DP, CL, and PS conceived the study. QM, DP, and CL designed the study. CL, DP, MO, MB, TC, MW, LB, and BG performed the experiments. CL, MB, DP, MA, and QM performed analyses. CL provided draft manuscript. QM finalized the manuscript. All authors contributed to manuscript preparation.

## Conflict of Interest

The authors declare that the research was conducted in the absence of any commercial or financial relationships that could be construed as a potential conflict of interest.

## Abbreviations

AA: arachidonic acid
ALOX5: arachidonate 5-lipoxigenase
ALOX5AP: arachidonate 5-lipoxigenase activating protein
ALOX15: arachidonate 15-lipoxigenase
AM: alveolar macrophage
AT: aspirin-triggered
BMDIM: bone marrow-derived inflammatory monocytes
C60F: fullerene-C60
CI: confidence interval
CNT: carbon nanotube
COX: cyclooxygenase
DHA: docosahexaenoic acid
ENM: engineered nanomaterial
EPA: eicosapentaenoic acid
FACS: fluorescence-activated cell sorting
FSC: forward scattering
H&E: hematoxylin and eosin
IFN: interferon
IL: interleukin
LC-MS-MS: liquid chromatography with tandem mass spectrometry
LM: lipid mediator
LPS: lipopolysaccharides
LT: leukotriene
LX: lipoxin
M1: classically activated macrophage
M2: alternatively activated macrophage
MWCNT: multi-walled carbon nanotube
PBS: phosphate buffered saline
PG: prostaglandin
PMN: polymorphonuclear leukocyte
RvD: resolvin D
RvE: resolvin E
RvT: resolving T
SPM: specialized pro-resolving mediator
SSC: side scattering
Th: T helper
TNF: tumor necrosis factor
TSLP: thymic stromal lymphopoietin
WLL: whole lung lavage.
